# Engineering of *Streptoalloteichus tenebrarius* 2444 for Sustainable Production of Tobramycin

**DOI:** 10.3390/molecules26144343

**Published:** 2021-07-18

**Authors:** Lena Mitousis, Hannes Maier, Luka Martinovic, Andreas Kulik, Sigrid Stockert, Wolfgang Wohlleben, Alfred Stiefel, Ewa M. Musiol-Kroll

**Affiliations:** 1 Interfaculty Institute of Microbiology and Infection Medicine (IMIT), Microbiology/Biotechnology, University of Tübingen, Auf der Morgenstelle 28, 72076 Tübingen, Germany; lena.mitousis@student.uni-tuebingen.de (L.M.); hannes.maier1995@gmail.com (H.M.); lukamartin1@aol.com (L.M.); andreas.kulik@uni-tuebingen.de (A.K.); sstockert@web.de (S.S.); wolfgang.wohlleben@biotech.uni-tuebingen.de (W.W.); 2 Cluster of Excellence ‘Controlling Microbes to Fight Infections’ (CMFI), University of Tübingen, Auf der Morgenstelle 28, 72076 Tübingen, Germany; 3 German Centre for Infection Research (DZIF), Partner site Tübingen, Auf der Morgenstelle 28, 72076 Tübingen, Germany; 4 Huvepharma EOOD/Biovet AD, 39 Petar Rakov Street, 4550 Peshtera, Bulgaria; Alfred.Stiefel@huvepharma.com

**Keywords:** actinomycetes, antibiotic, aminoglycoside, carbamoyltobramycin, tobramycin, apramycin, genetic engineering, yield improvement

## Abstract

Tobramycin is a broad-spectrum aminoglycoside antibiotic agent. The compound is obtained from the base-catalyzed hydrolysis of carbamoyltobramycin (CTB), which is naturally produced by the actinomycete *Streptoalloteichus tenebrarius*. However, the strain uses the same precursors to synthesize several structurally related aminoglycosides. Consequently, the production yields of tobramycin are low, and the compound’s purification is very challenging, costly, and time-consuming. In this study, the production of the main undesired product, apramycin, in the industrial isolate *Streptoalloteichus tenebrarius* 2444 was decreased by applying the fermentation media M10 and M11, which contained high concentrations of starch and dextrin. Furthermore, the strain was genetically engineered by the inactivation of the *aprK* gene (∆*aprK*), resulting in the abolishment of apramycin biosynthesis. In the next step of strain development, an additional copy of the tobramycin biosynthetic gene cluster (BGC) was introduced into the ∆*aprK* mutant. Fermentation by the engineered strain (*∆aprK*_1-17L) in M11 medium resulted in a 3- to 4-fold higher production than fermentation by the precursor strain (∆*aprK*). The phenotypic stability of the mutant without selection pressure was validated. The use of the engineered *S. tenebrarius* 2444 facilitates a step-saving, efficient, and, thus, more sustainable production of the valuable compound tobramycin on an industrial scale.

## 1. Introduction

Antibiotics, including the aminoglycoside tobramycin, are indispensable therapeutic tools of medicine. Tobramycin is very effective against Gram-negative bacteria such as *Pseudomonas aeruginosa*, and thus, the compound is used to treat severe infections that are particularly life-threatening for cystic fibrosis patients. The biological activity of tobramycin is mediated by its binding to the 30S subunit of the ribosome and subsequent inhibition of bacterial protein synthesis [1,2,3].

**Figure 1:**
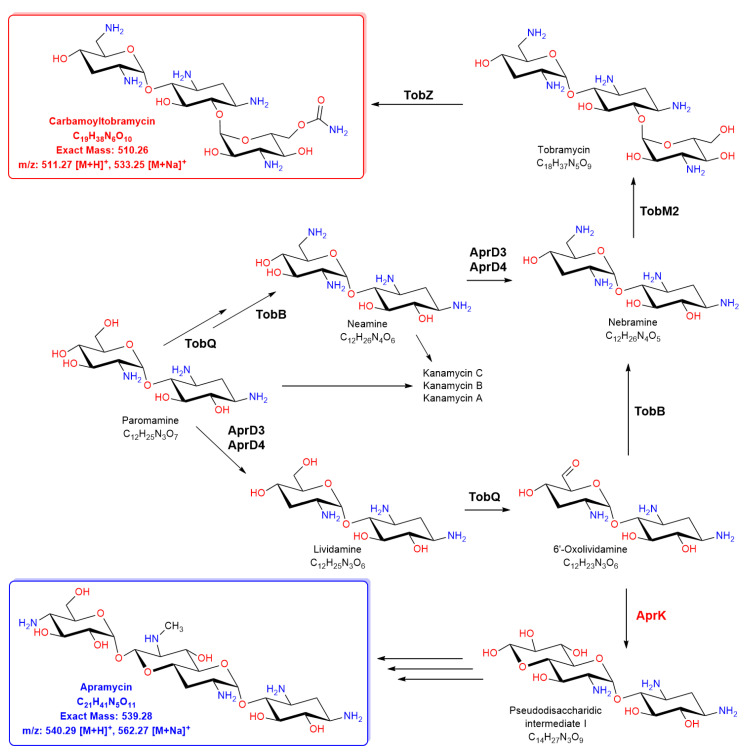
Proposed biosynthetic pathways for carbamoyltobramycin and apramycin (modified from Lv et al. [9] and Xiao et al. [10]). The biosynthesis starts with paromamine, which is the key branch point intermediate in aminoglycoside biosynthesis in *Streptoalloteichus tenebrarius*.

The structure of tobramycin features an aminocyclitol 2-deoxystreptamine (2-DOS) as a central aglycon in the pseudo-oligosaccharide moiety [4,5,6] (common pseudodisaccharide intermediates are paromamine and neamine) (Figure 1). In the case of tobramycin, the 2-DOS-hydroxyl groups are substituted with other aminocyclitols at positions 4 and 6. There are also aminoglycosides in which the 2-DOS-hydroxyl groups are substituted at positions 4 and 5 (e.g., ribostamycin, neomycin, hybrimycin, and butirosin). The 2-DOS structure is derived from d-glucose-6-phosphate via numerous enzymatic steps involving 2-deoxy-scyllo-inosose synthase, the dual-functional l-glutamine:2-DOI aminotransferase, and 2-deoxy-scyllo-inosamine dehydrogenase [7,8].

The antibiotic tobramycin is obtained from the base-catalyzed hydrolysis of carbamoyltobramycin (CTB), which is produced by *Streptoalloteichus tenebrarius* [11] (formerly “*Streptomyces tenebrarius*”, Higgens and Kastner, 1967 [12]). In general, the aminoglycoside producers synthesize many aminoglycoside congeners simultaneously. The sequencing of those strains and sequence analysis resulted in the identification of several aminoglycoside biosynthetic gene clusters (BGCs) [13] including the tobramycin BGC from *Streptoalloteichus tenebrarius* ATCC 17920 [14] and *S.* sp. (*tenebrarius*) DSM 40477 [8]. Using the BGC information, genetics, and biochemical approaches, it has been shown that parallel biosynthetic pathways are active during fermentation by the producer strains, and consequently, aminoglycoside congeners are present in the fermentation cultures [9,15,16]. For example, Lv et al. demonstrated in enzyme assays in vitro that the AprD4/AprD3 enzyme system acts on different pseudodisaccharide substrates in parallel pathways and is involved in the biosynthesis of CTB, apramycin, and other aminoglycosides (Figure 1) [9]. Interestingly, the disruption of the putative NDP-octodiose synthase gene *aprK* in *S. tenebrarius* ST316 abolished the apramycin biosynthesis and increased the CTB production [10]. On the other hand, the inactivation of *aprD3–D4* and *aprQ* in *Streptomyces tenebrarius* H6 (according to the current classification, *Streptoalloteichus tenebrarius*) resulted in a mutant that was blocked in apramycin and tobramycin biosynthesis. However, the production of the intermediates for the biosynthesis of kanamycin B increased [17].

Although genetic engineering has been conducted for some tobramycin producers, there are non-modified industrial strains, such as *Streptoalloteichus tenebrarius* 2444, in which the production is not optimized. Hence, the manufacture of tobramycin with this strain is very costly, and the downstream processing requires laborious purification steps to separate the aminoglycoside congeners. In this study, we applied different strategies, including medium screening, genetic manipulation, and BGC overexpression for the optimization of carbamoyltobramycin biosynthesis in the industrial isolate *S. tenebrarius* 2444.

## 2. Materials and Methods

### 2.1. Bacterial Strains and General Fermentation and Growth Conditions

The plasmids and strains used in this study are listed in the Supplementary Materials (Table S1.1–1.2 and Table S2). The *Streptoalloteichus tenebrarius* 2444 parental strain (PS) was obtained from the internal strain collection at the company Biovet (Huvepharma EOOD/Biovet AD Peshtera, Bulgaria). For routine cultivations of the parental strain and its mutants, tryptic soy broth (TSB) and amber-colored flasks (light-protection) were used. The compositions of all the screening media and other media are listed in the Supplementary Materials (Table S3). The standard propagation of the mutants was performed at 37 °C (180 rpm) in the presence of the respective antibiotics for selection (e.g., erythromycin at 100–150 μg/mL and/or thiostrepton at 150–200 μg/mL) (Supplementary Materials Table S4). *Escherichia coli* Stellar^TM^ Competent Cells (Takara Bio Europe, Saint-Germain-en-Laye, France) (Table S2) were used for the cloning procedure to obtain the pEM-constructs (Table S1.2). *E. coli* was cultivated at 37 °C (180 rpm) in lysogeny broth (LB) medium supplemented with appropriate antibiotics.

### 2.2. Genome Sequencing, Analysis, and Cloning of the aprK Gene Inactivation Construct

**Figure 2:**
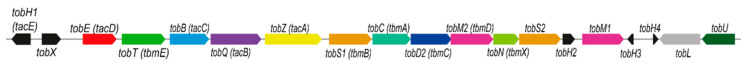
Tobramycin biosynthetic gene cluster (BGC) of *Streptoalloteichus tenebrarius* 2444 PS (https://www.dropbox.com/sh/jwxxdzh26worn7g/AADH7xfjNLeUB_V9MMBTYuuWa?dl=0). The National Center for Biotechnology Information (NCBI) database [21] contains tobramycin biosynthetic genes and proteins that include the prefix “*tob”*, “*tac”,* or “*tbm”*. Thus, different nomenclature exists for genes that encode identical proteins (e.g., *tobZ* (*tacA*)). In this overview, the “*tob”* abbreviation for the tobramycin BGC is preferred. The functions of the gene products are presented in Table 1.

The genomic DNA of *Streptoalloteichus tenebrarius* 2444 was isolated using a microbial DNA extraction kit (QIAGEN, Hilden, Germany). The genomic DNA samples were used for PacBio sequencing (Macrogen, PacBio sequencing procedure, Macrogen Inc., Seoul, South Korea). The draft genome was analyzed for the presence of biosynthetic gene clusters (BGCs) (Figure 2, Supplementary Materials Figures S1 and S2, Table 1 and Supplementary Materials Table S6) using the software antiSMASH [18] and Blast [19,20].

For the inactivation of the gene encoding a putative NDP-octodiose synthase (*aprK*) from the apramycin BGC, two fragments (upstream and downstream), flanking the gene *aprK,* were amplified in a PCR using the primers 115up_hldE3.REV/116up_hldE3.FOR and 113down_hldE3.FOR/114down_hldE3.REV, respectively. All the primer sequences used in this study are listed in the Supplementary Materials (Table S5). The upstream and downstream fragment of the *aprK* gene as well as an erythromycin resistance cassette (derived from pSP1 [26]) were cloned into a derivative of pGUSA21 [27] using the In-Fusion cloning kit (Takara Bio Europe). The generated construct was named pEM89. The Stellar^TM^ competent *E. coli* cells (Takara Bio Europe) were transformed according to the manufacturer’s specifications, including a 45 min recovery step in the super optimal broth with catabolite repression (S.O.C.) medium. Clones were selected on plates containing the appropriate antibiotics (Supplementary Materials Tables S1.2 and S4). For the isolation of plasmid DNA, *E. coli* clones were inoculated into and grew in liquid LB medium with the antibiotic for selection. The plasmid DNA was isolated using the PureYield^TM^ Plasmid Midiprep Kit (Promega, Madison, WI, USA) and sequenced (Eurofins Genomics, Ebersberg, Germany).

### 2.3. Generation of the aprK Gene Inactivation Construct

Competent cells of *E. coli* S17-1 (Supplementary Materials Table S2) were generated and subjected to calcium chloride transformation, yielding *E. coli* S17-1_pEM89. The S17-1 strain containing pEM89 was cultivated overnight in LB supplemented with chloramphenicol (100 μg/mL) and trimethoprim (10 μg/mL) at 37 °C (180 rpm). The overnight culture was used for the inoculation of a fresh culture (LB containing selection antibiotics) and let grow (37 °C, 180 rpm) until it reached an OD_600_ ~0.8. The cells were harvested and applied in intergeneric conjugation [28,29] to introduce the gene inactivation plasmid pEM89 into *Streptoalloteichus tenebrarius* 2444 PS. Single crossover mutants were isolated using MS (modified) agar plates (Supplementary Materials Table S3) containing the respective antibiotics for selection. The clones were isolated and cultivated at 37 °C for several generations on TSB medium. Sporulation agar [27] was used for the isolation of spores. Dilutions of the isolated spores were streaked out on plates containing the appropriate antibiotic (erythromycin at 100–150 μg/mL) for the selection of the double crossover mutants. The potential double crossover mutants were screened through control PCRs. The primers for the verification of the double crossovers are listed in the Supplementary Materials (Table S5).

### 2.4. Construction of the Tobramycin Cluster Overexpression Mutant

The construct pESAC-13 (*E. coli*–*Streptomyces* Artificial Chromosome containing oriT from the RK2 replicon, phiC31 integrase, phiC31 attP, an apramycin and thiostrepton resistance cassette) (Supplementary Materials Table S1.2) and DH10B *E. coli* cells (Bio S&T Inc., Québec, QC, Canada) were used for the generation of a PAC library of the *Streptoalloteichus tenebrarius* 2444 PS DNA. The inserts (average insert size > 115 Kb) were introduced into pESAC-13 via the BamHI cloning site. The PAC library was screened in a PCR using three primer pairs for the identification of the clones that carried the tobramycin BGC (P1_T_C_Fw/P2_T_C_Rev, P3_T_L_Fw/P4_T_L_Rev, and P5_T_R_Fw/P6_T_R_Rev) or the apramycin BGC (P7_A_C_Fw/P8_A_C_Rev, P9_A_L_Fw/P10_A_L_Rev, and P11_A_R_Fw/P12_A_R_Rev) (Supplementary Materials Table S5). The pESAC-13 containing the entire tobramycin BGC was modified by the insertion of an erythromycin resistance cassette using the Cre/lox-based recombination technology. The resulting PAC DNA (pESAC-13 with an erythromycin resistance cassette and the tobramycin cluster) was introduced and integrated into the genome of the ∆*aprK* mutant by conjugation. Clones containing an additional copy of the tobramycin BGC were selected on selection plates with erythromycin (150 μg/mL), thiostrepton (200 μg/mL), and nalidixic acid (50 μg/mL). The exconjugants were screened in control PCRs. The primers for the verification of the mutant containing an additional copy of the tobramycin BGC are listed in the Supplementary Materials (Table S5).

### 2.5. Production Assays

A pre-culture of *Streptoalloteichus tenebrarius* 2444 PS and/or its mutants was inoculated into TSB (first pre-culture). For that, 3 mL of a glycerol stock of the respective strain was added to 97 mL of TSB medium in amber-colored flasks containing nalidixic acid (25 μg/mL) (parental strain) and, in the case of the mutant strains, antibiotics for selection (erythromycin at 150 μg/mL and/or thiostrepton at 200 μg/mL). The first pre-culture was cultivated in a darkened rotary shaker (160 rpm) at 37 °C for 72 h. The cultures (approximately 20 mL) were centrifuged to determine the amount of pellet in each sample. The pellets were normalized by adding an appropriate amount of TSB medium to obtain the same cell density in each sample. For the inoculation of the second pre-culture, 10 mL of the normalized cell suspension was added to 90 mL of the production medium FC or screening medium (M9–M12, M14–M16) (Supplementary Materials Table S3). The second pre-culture was incubated in a darkened rotary shaker (160 rpm) at 37 °C for 48 h. Finally, triplicates of the main cultures were inoculated by adding 10 mL of the second pre-culture to 90 mL of the production medium FC or a screening medium (M9–M12, M14–M16). The fermentation took place in a darkened rotary shaker (160 rpm) at 30 °C for 168 h (7 days). The purity of the cultures and, in the case of the mutant strains, the stability of the genetic manipulations were examined by streaking a sample (200 µL) of the culture on TSB plates with and without selection antibiotics. In addition, samples (7 mL) were taken and centrifuged (10 min, 5000 rpm), and the supernatants were stored at −20 °C. The samples were thawed, and 2 mL of each sample was centrifuged (2 min, 13,000 rpm). Volumes of 1 mL were transferred into HPLC vials, and the samples were subjected to HPLC-MS.

The following conditions were applied for industrial fermentation (Biovet AD, Peshtera, Bulgaria): the first pre-culture was inoculated with mycelium of the respective strain grown on an TSB agar plate (1 cm^3^ piece of agar) and cultivated at 37 °C for 24 h (pH 7); the second pre-culture was inoculated with the first pre-culture (1:10 volume) and cultivated at 37 °C for 21 h (pH 6.8–6.3); the main culture was inoculated (1:10 volume) using the second pre-culture and cultivated at 37 °C for 120 h (pH and pO_2_—dissolved oxygen tension—were monitored). Feeding of glucose was conducted after 28 h and continued until the end of the fermentation. The pH in the main fermentation was maintained at 6.9–7.1. The value of pO_2lower_ (the minimum level of pO_2_) was 20% and it was maintained by applying an appropriate flow of aeriation gas and intensity of mixing.

### 2.6. HPLC-MS Analysis

The reference compounds (Supplementary Materials Figures S4 and S5) and samples of the production assays were analyzed on a HPLCLC/MSD Ultra Trap System XCT 6330 (Agilent Technologies, Santa Clara, CA, USA). As the stationary phase, a Gemini-110 C-18 column (5 µm, 150 × 2 mm + VS, column temperature 40 °C, Phenomenex, Torrance, CA, USA) was used. Solvent A (100% acetonitrile) and solvent B (0.7 mL/L ammonia solution, 25%, the pH adjusted with 1 N NaOH to 11.5) were used as the mobile phase, applying the gradient t_0_ = 100% B, t_9.5min_ = 70% B, t_10min_ = 5% B, stoptime 14 min, posttime 8 min of 100% solvent B (flow rate, 0.4 mL/min; injection volume, 2.5 μL). Data analysis was performed using the Agilent LC/MSD software ChemStation Rev. B.01.03, Agilent (Santa Clara, CA, USA). The following parameters were used for the MS detection, ionization: ESI positive and negative, alternating; mode: Ultra Scan; capillary voltage: 3.5 kV; temperature: 350 °C; target mass: *m*/*z* = 533 and *m*/*z* = 562. Data analyses were performed with the software 6300 Series Trap Control Version 6.1, Bruker Daltonik (Agilent, Santa Clara, CA, USA). The extracted ion chromatogram of the positive mode for *m*/*z* [M + Na] = 562 and *m*/*z* [M + Na] = 533 and the feature “smooth chromatogram” were applied to determine the peak area for apramycin (APRA, RT of approx. 2 min) and carbamoyltobramycin (CTB, RT of approx. 5.5 min), respectively. The area of the peak intensity for the apramycin and/or tobramycin mass (*m*/*z* [M + Na] = 562 and *m*/*z* [M + Na] = 533) was used for quantification.

### 2.7. Statistics Using Student’s t-Test

Inferential statistic (Student’s *t*-test) was applied to determine if there is a significant difference between the means of two sets of data. The Student’s *t*-test was performed using the software Excel. The *p*-values and the hypotheses for the Student’s *t*-test were included in Supplementary Materials (Tables S8 and S9).

## 3. Results and Discussion

### 3.1. Identification of the Tobramycin Biosynthetic Gene Cluster in Streptoalloteichus tenebrarius 2444

The genome of the strain *Streptoalloteichus tenebrarius* 2444 PS was sequenced and analyzed using the software antiSMASH [18]. In total, 33 putative BGCs were identified, including the apramycin (Supplementary Materials Figures S1 and S2, Table S6) and tobramycin BGC (Figure 2, Table 1). The tobramycin BGC of *S. tenebrarius* 2444 PS spans a region of approximately 22.2 kb of DNA with a GC content (% G + C) of 73%. The genes of the tobramycin BGC of *S. tenebrarius* 2444 PS were annotated using Prokka (v1.12b) [30]. The cluster consisted of 19 open reading frames (ORFs) (*tobH1*, *tobX*, *tobE*, *tobT*, *tobB*, *tobQ*, *tobZ*, *tobS1*, *tobC*, *tobD2*, *tobM2*, *tobN*, *tobS2*, *tobH2*, *tobM1*, *tobH3*, *tobH4*, *tobL*, and *tobU*) (Figure 2 and Table 1). The amino acid sequences of the gene products were analyzed using BlastP [19,20]. Except for TobM1, all the gene products (TobH1, TobX, TobE, TobT, TobB, TobQ, TobZ, TobS1, TobC, TobD2, TobM2, TobN, TobS2, TobH2, TobH3, TobH4, TobL, and TobU) were 100% identical to those previously described for a tobramycin BGC [13,31,32] in another *S. tenebrarius* strain (Table 1). The tobramycin BGC contains genes for biosynthesis and transport (resistance) as well as genes encoding proteins with uncharacterized function (Table 1). TobM1 (a putative aminoglycoside 4-glucosaminyltransferase) shows 99.76% identity to the respective gene product from *Streptoalloteichus tenebrarius* DSM 40477T. The divergence is caused by a different annotation of the *tobM1* gene in the genome of *S. tenebrarius* DSM 40477T and *S. tenebrarius* 2444 (Supplementary Materials Figure S3). Depending on the start codon of *tobM1*, GTG or ATG, the N-terminus of TobM1 may be extended by the amino acid (AA) sequence MGRGP. In the strains that use GTG as the start codon, a shorter version of TobM1 that is missing the five AAs on the N-terminus is generated. The analysis of the DNA sequence of *tobM1* revealed that the sequences were identical for both strains (*S. tenebrarius* DSM 40477T and *S. tenebrarius* 2444) in this region. It is very likely that the potential extension by the five N-terminal AAs MGRGP does not affect the function of TobM1 and that TobM1 acts as a putative aminoglycoside 4-glucosaminyltransferase in the biosynthesis of tobramycin, independently from the length of the putative protein.

### 3.2. Aminoglycoside Production in Streptoalloteichus tenebrarius 2444 PS

**Figure 3:**
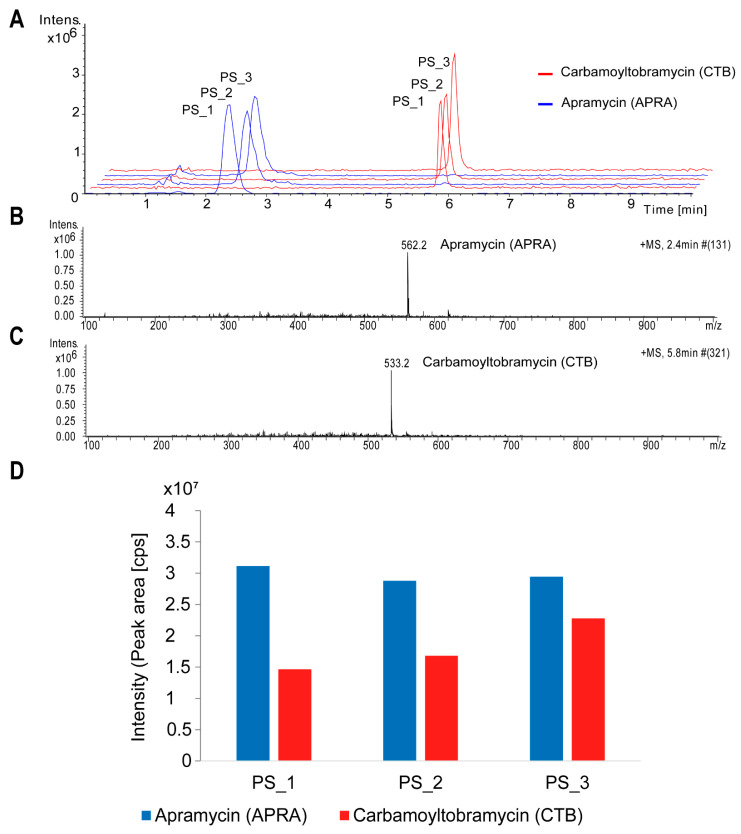
Apramycin (APRA) and carbamoyltobramycin (CTB) production in *Streptoalloteichus tenebrarius* 2444 PS (in triplicate, PS_1–3). (**A**) HPLC-ESI MS spectra for APRA and CTB. (**B**) Extracted ion chromatogram (EIC) for APRA (*m*/*z* 562, positive mode). (**C**) EIC for CTB (*m*/*z* 533, positive mode). (**D**) The intensity (peak area) for CTB and APRA mass peaks: cps, counts per second (the number of ions that hit the detector per unit of time).

To establish an aminoglycoside detection method and determine the aminoglycoside compounds produced by *Streptoalloteichus tenebrarius* 2444 PS, the strain was subjected to a production assay using the complex FC medium (which contained high amounts of soy flour, glucose, and salts such as NH_4_Cl and MgSO_4_; Supplementary Materials Table S3). Two major mass peaks of *m*/*z* 562 [M + Na]^+^ and *m*/*z* 533 [M + Na]^+^ were identified in the positive mode in the culture filtrate of *S. tenebrarius* 2444 PS (Figure 3). The retention time and mass of the compound were assigned to the aminoglycoside compounds apramycin (APRA) and carbamoyltobramycin (CTB), respectively (Figure 3, Supplementary Materials Figures S4 and S5). A triplet experiment demonstrated that *S. tenebrarius* 2444 PS produced more APRA compared with the production yields of CTB in FC medium (Figure 3D and Supplementary Materials Table S7).

**Figure 4:**
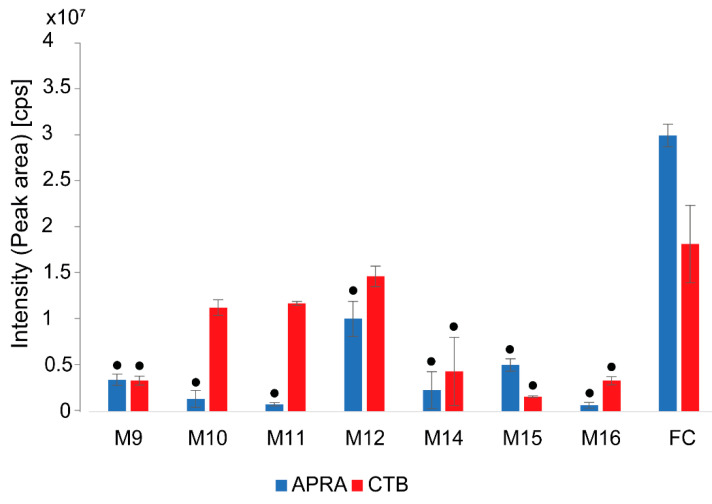
Production of apramycin (APRA) and carbamoyltobramycin (CTB) in Streptoalloteichus tenebrarius 2444 PS on different fermentation media: cps, counts per second (the number of ions that hit the detector per unit of time). The bars represent the means of triplicates (3 independent biological replicates), and the error bars represent the standard deviation (SD). The hypotheses H0 and H1 as well as the *p*-values of the Student’s *t*-test are included in Supplementary Materials Table S8. The results with *p*-values < 0.05 (production in each medium compared with the production in the FC medium) are marked with a black dot.

To optimize the CTB production and reduce the APRA yields, different media (M9–M12, M14–M16, and FC) (Figure 4) were tested in a screening approach. The amounts of APRA and CTB were determined for each sample of the screening (Figure 4 and Supplementary Materials Table S8). The results demonstrated that the production yields of APRA and CTB were generally lower in the media M9–M12 and M14–M16 than in the FC medium. Interestingly, depending on the medium, the APRA:CTB ratio was shifted towards either APRA or CTB. While the media M15 and FC supported the production of APRA, M10–M12, M14, and M16 were beneficial for CTB production and reduced the APRA yields in the culture. In the medium M9, very similar amounts of APRA and CTB were detected, which shows that an approximately 1:1 (APRA:CTB) ratio can be achieved by applying the appropriate medium. As the aim of this study was to increase CTB and reduce or eliminate APRA production, the most promising media for accomplishing this goal were M10 (starch, d-glucose, corn steep solids, soy flour, CaCO_3_, and CoCl_2_) and M11 (dextrin, d-glucose, soy flour, CaCO_3_, and CoCl_2_). In these media, the production of APRA was drastically reduced, while the CTB yields remained relatively high compared with those in the other tested media (Figure 4). Such carbon source-dependent aminoglycoside production was also observed by Nielsen and co-workers for *S. tenebrarius* cultivated on glucose and on an equimolar mixture of glucose and glycerol [33]. In their study, the amounts of kanamycin were decreased and those of apramycin increased, while the production yields of tobramycin and tobramycin carbamate remained unchanged, when a mixture of glucose and glycerol was used as a carbon source instead of glycerol in the minimal medium.

It is very likely that, in our work, the decrease in APRA production was caused by the starch and dextrin in the media M10 and M11, respectively. The other components (d-glucose, soy flour, CaCO_3_, and CoCl_2_) were also present in some of the tested media and did not shift the APRA:CTB ratio (Figure 4). Dextrin is a product of the hydrolysis of starch or glycogen, and thus, the compositions of M10 and M11 are similar, which is reflected in the production profiles obtained for these media (Figure 4). A positive effect of dextrin on the production of a sugar-containing antibiotic was described for neomycin [34] and spiramycin [35]. For the latter, which is a 16-membered macrolide, Yao et al. observed that the use of dextrin (obtained from a certain vendor) resulted in a twofold increase in spiramycin production in *Streptomyces ambofaciens*. Furthermore, the authors showed that the content of spiramycin by-products was affected, possibly due to the presence of dextrin in the medium and its influence on the expression of genes that encode enzymes (e.g., 4-aminotransferase) involved in forosamine and mycarose biosynthesis [35]. These sugar structures are attached to the aglycon of spiramycin. Like spiramycin, the biosynthesis of 2-deoxystreptamine (DOS)-containing aminoglycosides, such as tobramycin, requires aminotransferases. Therefore, dextrin might have a similar impact on the expression of genes (e.g., aminotransferase genes) involved in tobramycin biosynthesis.

### 3.3. Elimination of Apramycin Production by Genetic Engineering

The media M10 and M11 indeed shifted the APRA:CTB ratio in *Streptoalloteichus tenebrarius* 2444 PS; however, the goal of this engineering study was a decrease in or elimination of apramycin biosynthesis, so we strove to block apramycin biosynthesis. In a previous study [10], it was described that the gene *aprK* encoded a putative NDP-octodiose synthase that catalyzes important steps of the formation of the octodiose moiety in apramycin. To identify the crucial steps for the apramycin pathway in *S. tenebrarius* 2444 PS, the apramycin BGC was analyzed using the antiSMASH [18] and Blast tools [19,20] (Supplementary Materials Figures S1 and S2, Table S6). The putative apramycin BGC encompasses a region of ~38 kb (GC content of 73%) that contains 33 open reading frames (Supplementary Materials Figure S1 and Table S6). Most of the genes are identical to the previously described apramycin BGCs [8]. Differences were discovered in the AA sequences of AprJ (a putative phosphosugar mutase) and AprM (a putative glycosyltransferase) (Supplementary Materials Table S6 and Figure S2). Interestingly, the *aprM* gene is located directly upstream of the *aprK* gene in the apramycin BGC (Supplementary Materials Figure S1). The AA sequence of AprK from *S. tenebrarius* 2444 PS matches the AA sequence of the published AprK [10]. Therefore, we speculated that the AprK is also essential for the biosynthesis of the octodiose moiety of apramycin in *S. tenebrarius* 2444. However, it was not clear if the mutations in this region of the BGC (*aprJ* and *aprM*) might affect the apramycin pathway or the phenotype of a potential *aprK* deletion mutant.

**Figure 5:**
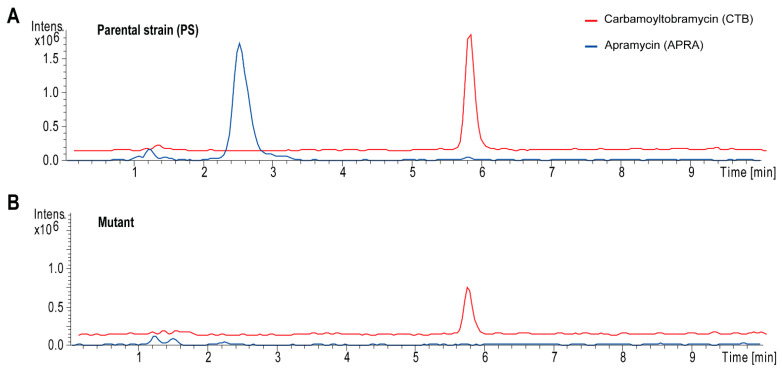
Extracted ion chromatograms for apramycin (APRA) (*m*/*z* [M + Na]^+^ = 562) and carbamoyltobramycin (CTB) (*m*/*z* [M + Na]^+^ = 533). (**A**) *Streptoalloteichus tenebrarius* 2444 parental strain (PS). (**B**) Gene knockout mutant ∆*aprK*.

To abolish apramycin biosynthesis, the construct pEM89 (Supplementary Materials Table S1.2) was cloned and used for the inactivation of the gene *aprK*. The pEM89 DNA was introduced into *S. tenebrarius* 2444, and single and double crossover mutants were isolated (Supplementary Materials Table S2). The *aprK* mutant, in which the gene was replaced by an erythromycin resistance cassette, was confirmed using screening PCRs (Supplementary Materials Figure S6). Subsequently, production assays with the *S. tenebrarius* 2444 PS and the *aprK* knockout mutant (∆*aprK*) were conducted in FC medium, and the samples were analyzed using HPLC-MS (Figure 5).

The analysis of the HPLC-MS spectra for the *S. tenebrarius* 2444 PS showed that the strain produced APRA (*m*/*z* 562 [M + Na]^+^) and CTB (*m*/*z* 533 [M + Na]^+^). The production of APRA was completely abolished in ∆*aprK* (Figure 5). The ∆*aprK* mutant retained the ability to produce CTB; however, it produced approximately 35% less than the *S. tenebrarius* 2444 PS (Figure 5). This contrasted with a previous observation, where an ~9% increase in CTB production was detected in the *aprK* mutant [10]. It is possible that mutations in the upstream and/or downstream region of the *aprK* gene in *S. tenebrarius* 2444 influenced the aminoglycoside biosynthesis in this strain and resulted in the lower CTB yields.

### 3.4. Overexpression of the Tobramycin Biosynthetic Gene Cluster

To further optimize the CTB production in the ∆*aprK* mutant strain, we aimed at the introduction of an additional copy of the tobramycin BGC in the APRA-null mutant strain. Therefore, a PAC library using the genomic DNA and pESAC-13 was generated. The PAC library clones were screened by PCR for the presence of the tobramycin BGC. For the clone 1-17L, PCR fragments of the correct size were amplified using the three primer pairs for the left, central, and right parts of the tobramycin BGC (Supplementary Materials Table S5 and Figure S7). This meant that the PAC contained the entire tobramycin BGC.

The PAC 1-17L, carrying the tobramycin gene cluster, was introduced into the ∆*aprK* mutant using conjugation and integrated into the mutant’s genome (Supplementary Materials Tables S1.2 and S2 and Figure S8). In parallel with the cluster overexpression approach, the DNA of the 1-17L PAC was transferred into diverse *Streptomyces* hosts (*Streptomyces coelicolor* M1146, M1152, and M1154; *Streptomyces lividans* TK24; and *Streptomyces albus*) for heterologous production of tobramycin (data not shown), an alternative strategy for the elimination of the unwanted product (apramycin). A further advantage of this undertaking was the fact that standard protocols for genetic manipulation were available for the heterologous hosts. This would enable an easy and fast improvement of the production yields, in the case of successful tobramycin production in such heterologous systems. However, neither CTB nor tobramycin was detected in the culture supernatants and extracts obtained from the strains used for the heterologous expression of the tobramycin BGC (data not shown). This suggests that either the BGC was not expressed, or important genes/enzymes are missing in the tested hosts. The latter is supported by the fact that genes/enzymes from parallel aminoglycoside pathways are required for the biosynthesis of these compounds, as has been demonstrated by Park et al. for the aminoglycoside kanamycin [16].

**Figure 6:**
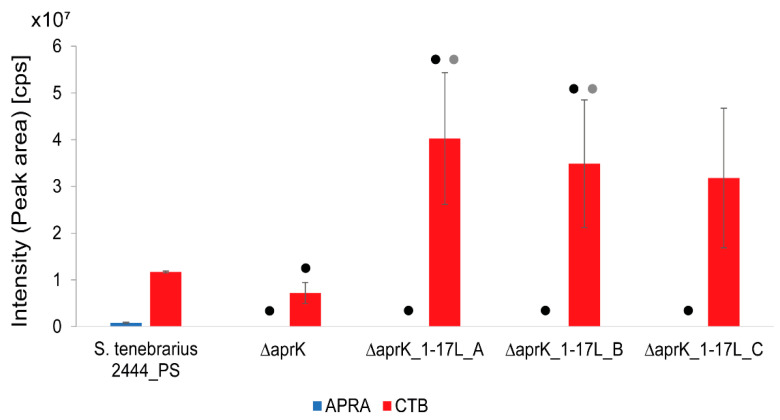
Apramycin (APRA) and carbamoyltobramycin (CTB) production in the *Streptoalloteichus tenebrarius* 2444 PS, the ∆*aprK* mutant, and the ∆*aprK* mutant containing an additional copy of the tobramycin BGC (triplicates for three isolates of ∆*aprK*_1-17L, **A**–**C**). The production assay was conducted in M11 medium. cps, counts per second (the number of ions that hit the detector per unit of time). The bars represent the means of triplicates (3 independent biological replicates), and the error bars represent the standard deviation (SD). The hypotheses H0 and H1 as well as the *p*-values of the Student’s *t*-test are included in Supplementary Materials Table S9. The results with *p*-values < 0.05 (each sample compared with *S. tenebrarius* 2444 PS) are marked with a black dot. The results with *p*-values < 0.05 (each sample compared with the ∆*aprK* mutant) are marked with a grey dot.

In parallel with the heterologous expression strains, three isolates of the ∆*aprK*_1-17L mutant were subjected to production assays. The results of the HPLC-MS analysis demonstrated that the CTB production in ∆*aprK*_1-17L (which contained two copies of the tobramycin BGC) was approximately 3–4 times higher than the CTB production in the precursor strain (∆*aprK*) in M11 medium (Figure 6 and Supplementary Materials Table S9). The standard deviation for the production in the three isolates was relatively high, which might have been caused by variations in the growth (e.g., generation of cell aggregates) of each culture. However, the mean values of the triplicates were similar. Therefore, our data strongly indicate that the level of CTB depends on the copy number of the tobramycin BGC. A similar relation, in which the production was dependent on the copy number of the respective BGC, was reported by Bibb and co-workers for the aminoglycoside kanamycin [36]. The titer of a control strain containing a single copy of the kanamycin BGC was lower than that of a strain with two copies of the cluster. Positive effects of a cluster duplication on the production yields were also observed for polyketides and a peptidyl nucleoside antibiotic such as tautomycetin from *Streptomyces* sp. CK4412 [37] (14-fold enhanced productivity), spinosyn from *Saccharopolyspora spinosa* [38], and nikkomycin from *Streptomyces ansochromogenes* [39].

### 3.5. Effect of Selection Antibiotics

As the addition of selection antibiotics for large-scale fermentation and manufacture of tobramycin using recombinant mutant strains would require additional purification steps for removing the selection antibiotics from the final product, we aimed at the generation of genetically stable engineered strains and a fermentation free of selection antibiotics.

In fact, the reversion of the ∆*aprK* mutant is not possible, as the gene was replaced by the erythromycin resistance cassette. However, the additional copy of the tobramycin BGC is integrated into the genome and could be lost via disintegration. This would lead to phenotypic instability in the fermentation.

To test the phenotypic stability of the generated mutants, the precursor strain ∆*aprK* and three isolates of the ∆*aprK*_1-17L cluster overexpression mutant were subjected to production assays. The assays were conducted in M11 medium with and without the respective selection antibiotics over the entire fermentation process (two pre-cultures and a main culture) (Section 2.5 in Material and Methods). Samples of the main fermentation culture were taken for streaking on TSB-agar plates containing the selection antibiotics as well as for HPLC-MS analysis. All the strains showed comparable density and growth on the TSB-agar plates with and without the respective selection antibiotics, which strongly indicated that the mutants were genetically stable. In contrast to many replicative plasmids [40,41], the genetic engineering strategy applied in this study ensured a better stability of the recombinant strains.

**Figure 7:**
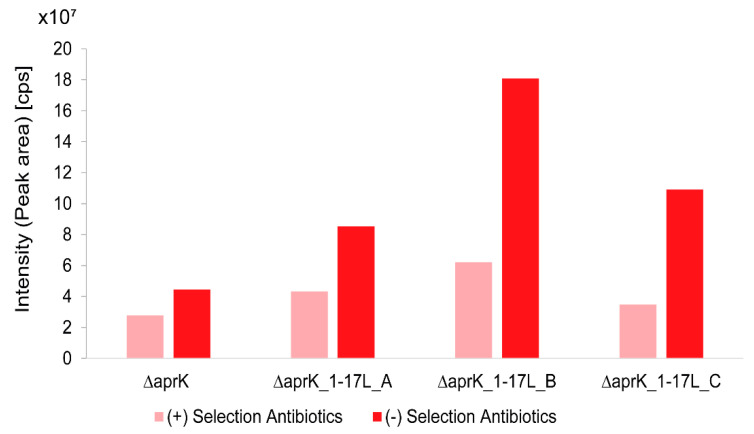
Production of carbamoyltobramycin (CTB) in the presence and absence of selection antibiotics in M11 medium. The gene knockout mutant ∆*aprK* and three isolates of ∆*aprK*_1-17L (**A**–**C**) were analyzed. cps, counts per second (the number of ions that hit the detector per unit of time).

The evaluation of the HPLC-MS data showed that the production of CTB in cultures without the selection antibiotics was 1–3 times higher than the yields observed for cultures of the same strain that contained the selection antibiotics (Figure 7 and Supplementary Materials Table S10). These results revealed that the selection antibiotics negatively influenced the CTB production.

To verify the genetic stability of producer strains and reproducibility of the production yields, the mutants generated in this study were examined under industrial fermentation conditions (Section 2.5 in Material and Methods) without selection antibiotics in the main production culture. The data confirmed the results obtained at the laboratory scale. Thus, the mutants generated in this study are suitable for the industrial manufacture of tobramycin.

## 4. Conclusions

*Streptoalloteichus tenebrarius* 2444 PS is an industrial isolate that produces a mixture of similar aminoglycosides. This is very challenging for the industrial production of tobramycin. Several steps are necessary for the purification of the final product, tobramycin.

Using genetic engineering approaches, we successfully modified the strain for an optimized production of tobramycin. More specifically, a gene (*aprK*) essential for the biosynthesis of a competitive product (apramycin) was inactivated by its replacement with an erythromycin resistance cassette. Moreover, a second copy of the tobramycin BGC was introduced into the genome of the ∆*aprK* mutant. The obtained mutant is not only blocked in the biosynthesis of the main unwanted by-product, but it also increased the production yields (3- to 4-fold) of carbamoyltobramycin, which is used for the synthesis of the final product, tobramycin.

Particularly advantageous is the fact that the gene knockout and integration of an additional copy of the tobramycin BGC do not require the addition of selection antibiotics for the stable production of the aminoglycoside antibiotic under industrial fermentation conditions. A clone of the cluster overexpression mutants was selected and approved as a new production strain for the manufacture of tobramycin.

Therefore, the strategy presented in this study can be applied to optimize producer strains for more sustainable production of antibiotics and other valuable bioactive compounds.

## Figures and Tables

**Table 1:** Proposed functions of the products of genes encoded in the tobramycin biosynthetic gene cluster of *Streptoalloteichus tenebrarius* 2444 PS.

Gene	Protein	BlastP Hits (Accession)	Percent Identity (BlastP)	Putative Function	References for Functional Characterization
*tobH1* (*tacE*)	*TobH1* (*tacE*)	TobH1 (TacE) (CAH18547.1)	100%	Unknown	-
		YvrE (SHG81534.1)	79.59%	Sugar lactone lactonase YvrE	-
*tobX*	TobX	TobX (CAH18549.1)	100%	Unknown (dioxygenase)	-
*tobE* (*tacD*)	TobE (TacD)	TobE (Q2MF22.1)	100%	2-deoxy-scyllo-inosamine dehydrogenase (DOIA dehydrogenase)	-
*tobT* (*tbmE*)	TobT (TbmE)	TbmE (CAE22476.1)	100%	Major facilitator superfamily protein	-
*tobB* (*tacC*)	TobB (TacC)	Hypothetical protein (CAE22475.1)	100%	Unknown	-
		Hypothetical protein (WP_073480773.1)	86.84%	Aminotransferase class III-fold pyridoxal phosphate-dependent enzyme	-
*tobQ* (*tacB*)	TobQ (TacB)	Hypothetical protein (CAE22474.1)	100%	6′-dehydrogenase	[22]
		Hypothetical protein (WP_073480774.1)	89.37%	GMC family oxidoreductase	-
*tobZ* (*tacA*)	TobZ (TacA)	TacA (CAE22473.1) (Q70IY1.1)	100%	O-carbamoyltransferase	[10,23]
*tobS1* (*tbmB*)	TobS1 (TbmB)	TbmB (Q2MF17.1)	100%	2-deoxy-scyllo-inosose aminotransferase (l-glutamine:DOI aminotransferase)	[24,25]
*tobC* (*tbmA*)	TobC (TbmA)	TbmA (Q2MF16.1)	100%	2-deoxy-scyllo-inosose synthase (DOI synthase)	[14,25]
*tobD2* (*tbmC*)	TobD2 (TbmC)	TbmC (CAE22470.1)	100%	Dehydrogenase	-
*tobM2* (*tbmD*)	TobM2 (TbmD)	TbmD (CAE22469.1)	100%	6-glycosyltransferase	[16]
*tobN* (*tbmX*)	TobN (TbmX)	TobN (CAH18559.1)	100%	Amidase	-
*tobS2*	TobS2	TobS2 (Q2MF12.1)	100%	l-glutamine:3-amino-2,3-dideoxy-scyllo-inosose aminotransferase (l-glutamine:amino-DOI aminotransferase)	-
*tobH2*	TobH2	Hypothetical protein (CAH18561.1)	100%	Retrotransposon protein (possibly non-functional)	-
*tobM1*	TobM1	TobM1 (CAH18562.1)	99.76%	Aminoglycoside 4-glucosaminyltransferase	[16]
*tobH3*	TobH3	No hits	-	Unknown	-
*tobH4*	TobH4	No hits	-	Unknown	-
*tobL*	TobL	TobL (CAH18563.1)	100%	Carbamoyl-phosphate synthase	-

## Data Availability

The sequences of the apramycin and tobramycin biosynthetic gene cluster (BGC) from *Streptoalloteichus tenebrarius* 2444 PS are openly available. The sequences were deposited in the Cloud (Dropbox storage service) and are accessible by using the link: https://www.dropbox.com/sh/jwxxdzh26worn7g/AADH7xfjNLeUB_V9MMBTYuuWa?dl=0. If you experience difficulties in accessing the data, please contact the corresponding author (ewa.musiol@biotech.uni-tuebingen.de) or molecules@mdpi.com.

## Supplementary Materials

Table S1: Synthetic fragment used in this study, Table S1.2: Plasmids, Table S2: Strains, Table S3: Media, Table S4: Antibiotics, Table S5: Primers and PCRs, Table S6: Analysis of the gene products of the genomic area covering the apramycin biosynthetic gene cluster of *Streptoalloteichus tenebrarius* 2444, Table S7: Raw data for carbamoyltobramycin (CTB) and apramycin (APRA) production in *S. tenebrarius* 2444 parental strain (PS), Table S8: Raw data for the medium screening, Table S9: Raw data for the strain carrying an additional copy of the tobramycin BGC, Table S10: Raw data for cultivation in presence (+) and absence (-) of selection antibiotics, Figure S1: Putative apramycin biosynthetic gene cluster (BGC) from *Streptoalloteichus tenebrarius* 2444, Figure S2: BlastP analysis of AprJ and AprM, Figure S3: BlastP analysis of TobM1, Figure S4. Spectra of the HPLC-MS analysis of the reference compound apramycin (APRA), Figure S5. Spectra of the HPLC-MS analysis of the reference compound carbamoyltobramycin (CTB), Figure S6. Analysis of screening PCRs for verification of the mutant ∆*aprK*, Figure S7: Identification of the PAC containing the tobramycin BGC, Figure S8: Introduction of the PAC containing the tobramycin BGC (1-17L) into the ∆*aprK* mutant.

## Sample Availability

Samples of the compounds apramycin and carbamoyltobramycin are available from the corresponding author.

## References

[1] Fosso M.Y., Zhu H., Green K.D., Garneau-Tsodikova S., Fredrick K. (2015). Tobramycin Variants with Enhanced Ribosome-Targeting Activity. ChemBioChem.

[2] Wasserman M.R., Pulk A., Zhou Z., Altman R.B., Zinder J.C., Green K.D., Garneau-Tsodikova S., Cate J.H., Blanchard S.C. (2015). Chemically Related 4,5-Linked Aminoglycoside Antibiotics Drive Subunit Rotation in Opposite Directions. Nat. Commun..

[3] Wang L., Pulk A., Wasserman M.R., Feldman M.B., Altman R.B., Cate J.H., Blanchard S.C. (2012). Allosteric Control of the Ribosome by Small-Molecule Antibiotics. Nat. Struct. Mol. Biol..

[4] Arya D.P. (2007). Aminoglycoside Antibiotics: From Chemical Biology to Drug Discovery.

[5] Benveniste R., Davies J. (1973). Structure-Activity Relationships among the Aminoglycoside Antibiotics: Role of Hydroxyl and Amino Groups. Antimicrob. Agents Chemother..

[6] Kudo F., Eguchi T. (2016). Aminoglycoside Antibiotics: New Insights into the Biosynthetic Machinery of Old Drugs. Chem. Rec..

[7] Llewellyn N.M., Spencer J.B. (2006). Biosynthesis of 2-Deoxystreptamine-Containing Aminoglycoside Antibiotics. Nat. Prod. Rep..

[8] Piepersberg W., Aboshanab K.M., Schmidt-Beißner H., Wehmeier U.F., Arya D.P. (2007). The Biochemistry and Genetics of Aminoglycoside Producers. Aminoglycoside Antibiotics: From Chemical Biology to Drug Discovery.

[9] Lv M., Ji X., Zhao J., Li Y., Zhang C., Su L., Ding W., Deng Z., Yu Y., Zhang Q. (2016). Characterization of a C3 Deoxygenation Pathway Reveals a Key Branch Point in Aminoglycoside Biosynthesis. J. Am. Chem. Soc..

[10] Xiao J., Li H., Wen S., Hong W. (2014). Concentrated Biosynthesis of Tobramycin by Genetically Engineered *Streptomyces tenebrarius*. J. Gen. Appl. Microbiol..

[11] Koch K., Davis F., Rhoades J. (1973). Nebramycin: Separation of the Complex and Identification of Factors 4, 5, and 5′. J. Antibiot..

[12] Tamura T., Ishida Y., Otoguro M., Hatano K., Suzuki K.-I. (2008). Classification of ‘*Streptomyces tenebrarius*’ Higgins and Kastner as *Streptoalloteichus tenebrarius* nom. rev., comb. nov., and Emended Description of the Genus *Streptoalloteichus*. Int. J. Syst. Evol. Microbiol..

[13] Kudo F., Eguchi T. (2009). Biosynthetic Genes for Aminoglycoside Antibiotics. J. Antibiot..

[14] Kharel M.K., Basnet D.B., Lee H.C., Liou K., Woo J.S., Kim B.-G., Sohng J.K. (2004). Isolation and Characterization of the Tobramycin Biosynthetic Gene Cluster from *Streptomyces tenebrarius*. FEMS Microbiol. Lett..

[15] Guo J., Huang F., Huang C., Duan X., Jian X., Leeper F., Deng Z., Leadlay P.F., Sun Y. (2014). Specificity and Promiscuity at the Branch Point in Gentamicin Biosynthesis. Chem. Biol..

[16] Park J.W., Park S.R., Nepal K.K., Han A.R., Ban Y.H., Yoo Y.J., Kim E.J., Kim E.M., Kim D., Sohng J.K. (2011). Discovery of Parallel Pathways of Kanamycin Biosynthesis Allows Antibiotic Manipulation. Nat. Chem. Biol..

[17] Ni X., Li D., Yang L., Huang T., Li H., Xia H. (2011). Construction of Kanamycin B Overproducing Strain by Genetic Engineering of *Streptomyces tenebrarius*. Appl. Microbiol. Biotechnol..

[18] Weber T., Blin K., Duddela S., Krug D., Kim H.U., Bruccoleri R., Lee S.Y., Fischbach M.A., Müller R., Wohlleben W. (2015). AntiSMASH 3.0—A Comprehensive Resource for the Genome Mining of Biosynthetic Gene Clusters. Nucleic Acids Res..

[19] Altschul S.F., Gish W., Miller W., Myers E.W., Lipman D.J. (1990). Basic Local Alignment Search Tool. J. Mol. Biol..

[20] Gish W., States D.J. (1993). Identification of Protein Coding Regions by Database Similarity Search. Nat. Genet..

[21] National Center for Biotechnology Information (NCBI). https://www.ncbi.nlm.nih.gov/.

[22] Yu Y., Hou X., Ni X., Xia H. (2008). Biosynthesis of 3′-Deoxy-Carbamoylkanamycin C in a *Streptomyces tenebrarius* Mutant Strain by *tacB* Gene Disruption. J. Antibiot..

[23] Parthier C., Görlich S., Jaenecke F., Breithaupt C., Bräuer U., Fandrich U., Clausnitzer D., Wehmeier U.F., Böttcher C., Scheel D. (2012). The O-Carbamoyltransferase TobZ Catalyzes an Ancient Enzymatic Reaction. Angew. Chem. Int. Ed. Engl..

[24] Kharel M.K., Subba B., Lee H.C., Liou K., Sohng J.K. (2005). Characterization of L-Glutamine: 2-Deoxy-Scyllo-Inosose Aminotransferase (*tbmB*) from *Streptomyces tenebrarius*. Bioorg. Med. Chem. Lett..

[25] Lin Y.-S., Hong W.-R. (2012). Construction of *Streptomyces tenebrarius* Mutant with Knockout of *tobS 1-C* Genes. J. China Pharm. Univ..

[26] Pelzer S., Reichert W., Huppert M., Heckmann D., Wohlleben W. (1997). Cloning and Analysis of a Peptide Synthetase Gene of the Balhimycin Producer *Amycolatopsis mediterranei* DSM5908 and Development of a Gene Disruption/Replacement System. J. Biotechnol..

[27] Robertsen H.L., Musiol-Kroll E.M., Ding L., Laiple K.J., Hofeditz T., Wohlleben W., Lee S.Y., Grond S., Weber T. (2018). Filling the Gaps in the Kirromycin Biosynthesis: Deciphering the Role of Genes Involved in Ethylmalonyl-CoA Supply and Tailoring Reactions. Sci. Rep..

[28] Kieser T., Bibb M.J., Buttner M.J., Chater K.F., Hopwood D.A. (2000). Practical Streptomyces Genetics.

[29] Musiol E.M., Greule A., Härtner T., Kulik A., Wohlleben W., Weber T. (2013). The AT2 Domain of KirCI Loads Malonyl Extender Units to the ACPs of the Kirromycin PKS. ChemBioChem.

[30] Seemann T. (2014). Prokka: Rapid Prokaryotic Genome Annotation. Bioinform.

[31] Flatt P.M., Mahmud T. (2007). Biosynthesis of Aminocyclitol-Aminoglycoside Antibiotics and Related Compounds. Nat. Prod. Rep..

[32] Kharel M.K., Subba B., Lee H.C., Liou K., Woo J.S., Sohng J.K. (2003). An Approach for Cloning Biosynthetic Genes of 2-Deoxystreptamine-Containing Aminocyclitol Antibiotics: Isolation of a Biosynthetic Gene Cluster of Tobramycin from *Streptomyces tenebrarius*. Biotechnol. Lett..

[33] Borodina I., Schöller C., Eliasson A., Nielsen J. (2005). Metabolic Network Analysis of *Streptomyces tenebrarius*, a *Streptomyces* Species with an Active Entner-Doudoroff Pathway. Appl. Environ. Microbiol..

[34] Majumdar M.K., Majumdar S. (1967). Utilization of Carbon-and Nitrogen-Containing Compounds for Neomycin Production by *Streptomyces fradiae*. Appl. Microbiol..

[35] Yao K., Gao S., Wu Y., Zhao Z., Wang W., Mao Q. (2018). Influence of Dextrins on the Production of Spiramycin and Impurity Components by *Streptomyces ambofaciens*. Folia Microbiol..

[36] Yanai K., Murakami T., Bibb M. (2006). Amplification of the Entire Kanamycin Biosynthetic Gene Cluster during Empirical Strain Improvement of *Streptomyces kanamyceticus*. Proc. Natl. Acad. Sci. USA.

[37] Nah H.-J., Woo M.-W., Choi S.-S., Kim E.-S. (2015). Precise Cloning and Tandem Integration of Large Polyketide Biosynthetic Gene Cluster Using *Streptomyces* Artificial Chromosome System. Microb. Cell Fact..

[38] Tang Y., Xia L., Ding X., Luo Y., Huang F., Jiang Y. (2011). Duplication of Partial Spinosyn Biosynthetic Gene Cluster in *Saccharopolyspora spinosa* Enhances Spinosyn Production. FEMS Microbiol. Lett..

[39] Liao G., Li J., Li L., Yang H., Tian Y., Tan H. (2010). Cloning, Reassembling and Integration of the Entire Nikkomycin Biosynthetic Gene Cluster into *Streptomyces ansochromogenes* Lead to an Improved Nikkomycin Production. Microb. Cell Fact..

[40] Thoma L., Sepulveda E., Latus A., Muth G. (2014). The Stability Region of the *Streptomyces lividans* plasmid pIJ101 Encodes a DNA-Binding Protein Recognizing a Highly Conserved Short Palindromic Sequence Motif. Front. Microbiol..

[41] De Gelder L., Ponciano J.M., Joyce P., Top E.M. (2007). Stability of a Promiscuous Plasmid in Different Hosts: No Guarantee for a Long-Term Relationship. Microbiology.

