# Supplementary material for: Engineering of Streptoalloteichus tenebrarius 2444 for Sustainable Production of Tobramycin
Source: Molecules. 2021 Jul 18;26(14):4343. doi: 10.3390/molecules26144343 (PMC8304502; doi:10.3390/molecules26144343)
Supplement: Supplementary file 1 [file molecules-26-04343-s001.zip › Supplementary Materials/molecules-1281617-supplementary.pdf]

Supplementary Materials

Engineering of *Streptoalloteichus tenebrarius* 2444 for Sustainable Production of Tobramycin

Lena Mitousis[1,2], Hannes Maier[1,2,3], Luka Martinovic[1,2], Andreas Kulik[1,2], Sigrid Stockert[1,2,†], Wolfgang Wohlleben[1,2,3], Alfred Stiefel[4], Ewa M. Musiol-Kroll[1,2,*]

[1]Interfaculty Institute of Microbiology and Infection Medicine (IMIT), Microbiology/Biotechnology, University of Tübingen, Auf der Morgenstelle 28, 72076 Tübingen, Germany

[2]Cluster of Excellence 'Controlling Microbes to Fight Infections' (CMFI), University of Tübingen, Auf der Morgenstelle 28, 72076 Tübingen, Germany

[3]German Centre for Infection Research (DZIF), Partner site Tübingen, Auf der Morgenstelle 28, 72076 Tübingen, Germany

[4]Huvepharma EOOD / Biovet AD, 39 Petar Rakov Street, 4550 Peshtera, Bulgaria

*Correspondence: ewa.musiol@biotech.uni-tuebingen.de

[5]Present address: Interfaculty Institute of Microbiology and Infection Medicine (IMIT), Department of Microbial Bioactive Compounds, University of Tübingen, Auf der Morgenstelle 28, 72076 Tübingen, Germany

Table of Contents

1. Supplementary materials and methods

- Synthetic fragment

- Plasmids

- Strains

- Media

- Antibiotics

- Primers and PCRs

2. Supplementary results

- Putative apramycin biosynthetic gene cluster (BGC) from *Streptoalloteichus tenebrarius* 2444

- Analysis of the gene products of the apramycin (BGC) of *Streptoalloteichus tenebrarius* 2444

- BlastP analysis of AprJ and AprM

- BlastP analysis of TobM1

- HPLC-MS analysis of the reference compounds (apramycin (APRA) and carbamoyltobramycin (CTB)

- Gene knockout mutant (Δ*aprK*)

- Identification of the PAC containing the tobramycin BGC

- Introduction of the PAC containing the tobramycin BGC (1-17L) into the Δ*aprK* mutant

- HPLC-MS analysis (raw data)

3. References

1. Supplementary materials and methods

Table S1.1: Synthetic fragment used in this study

Synthetic fragment	Specifications	Reference or source
CM^R-cassette	*cat promoter and chloramphenicol resistance cassette*, for cloning of pEM70*	GenScript

*Sequence:
ACGTCTAAGAAACCATTATTATCATGACATTAACCTATAAAAATAGGCGTATCACGAGGCCCT
TTCGTCTTCGAATAAATACCTGTGACGGAAGATCACTTCGCAGAATAAATAAATCCTGGTGTC
CCTGTTGATACCGGGAAGCCCTGGGCCAACTTTTGGCGAAAATGAGACGTTGATCGGCACGT
AAGAGGTTCCAACTTTCACCATAATGAAATAAGATCACTACCGGGCGTATTTTTTGAGTTATC
GAGATTTTCAGGAGCTAAGGAAGCTAAAATGGAGAAAAAAATCACTGGATATACCACCGTTG
ATATATCCCAATGGCATCGTAAAGAACATTTTGAGGCATTTCAGTCAGTTGCTCAATGTACCT
ATAACCAGACCGTTCAGCTGGATATTACGGCCTTTTTAAAGACCGTAAAGAAAAATAAGCAC
AAGTTTTATCCGGCCTTTATTCACATTCTTGCCCGCCTGATGAATGCTCATCCGGAATTCCGTA
TGGCAATGAAAGACGGTGAGCTGGTGATATGGGATAGTGTTCACCCTTGTTACACCGTTTTCC
ATGAGCAAACTGAAACGTTTTCATCGCTCTGGAGTGAATACCACGACGATTTCCGGCAGTTTC
TACACATATATTCGCAAGATGTGGCGTGTTACGGTGAAAACCTGGCCTATTTCCCTAAAGGGT
TTATTGAGAATATGTTTTTCGTCTCAGCCAATCCCTGGGTGAGTTTCACCAGTTTTGATTTAAAC
GTGGCCAATATGGACAACTTCTTCGCCCCCGTTTTCACCATGGGCAAATATTATACGCAAGGC
GACAAGGTGCTGATGCCGCTGGCGATTCAGGTTCATCATGCCGTTTGTGATGGCTTCCATGTCG
GCAGAATGCTTAATGAATTACAACAGTACTGCGATGAGTGGCAGGGCGGGGCGTAA

Table S1.2: Plasmids

Plasmid	Specifications	Reference or source
pGUSA21	*pSETGUS with gusA, Δint, ΔattB, MCS from pUC21, apramycin^R*	[1,2]
pEM70	*pGUSA21 derivative with synthetic CM^R-cassette (Table S1.1) which was cloned into the plasmid using the XhoI and XbaI restrictions sites*	This study
pre_pEM77	*pEM70 derivative with flanking regions (left and right fragments) of the gene aprK from the apramycin biosynthetic gene cluster (Streptoalloteichus tenebrarius 2444), The flanking regions were cloned using the I-SceI and BamHI restriction sites and the InFusion cloning kit*	This study
pEM77	*pre_pEM77 derivative with a thiostrepton ^{R}-cassette from pGM190* [3], *the thiostrepton ^{R}-cassette was cloned between the flanking regions of aprK using the XbaI restriction site and InFusion cloning kit*	This study
pEM89	*pEM77 derivative in which the thiostrepton ^{R}-cassette was replaced by erythromycin ^{R}-cassette from the plasmid pSP1* [4], *the*	This study

Plasmid	Specifications	Reference or source
	erythromycin ^{R}-cassette was cloned into the plasmid using the BglII and XbaI restriction site and InFusion cloning kit	
pESAC-13	*E. coli-Streptomyces Artificial Chromosome, pPAC-S1 derivative [5] containing oriT from the RK2 replicon, phiC31 integrase, phiC31 attP, an apramycin, and thiostrepton resistance cassette)*	(Bio S&T Inc.)
1-17L	*pESAC-13 derivative containing the entire tobramycin biosynthetic gene cluster (BGC)*	This study

Table S2: Strains

Strain	Specifications	Reference or source
***Escherichia coli* strains**		
Stellar™ Competent Cells (*E. coli* HST08)	*F-, endA1, supE44, thi-1, recA1, relA1, gyrA96, phoA, Φ80d lacZΔ M15, Δ(lacZYA-argF) U169, Δ(mrr-hsdRMS-mcrBC), ΔmcrA, λ-*	Takara Bio USA, Inc.®
S17-1 λpir	*recA, thi, pro, hsdR-M+RP4: 2-Tc:Mu: Km, Tn7 λpir, TpR, SmR*	Biomedal S.L.®
S17-1_pEM89	*Derivative of S17-1 λpir containg the plasmid pEM89*	This study
Actinomycetes strains		
Streptoalloteichus tenebrarius 2444	*Aminoglycoside producer (main products apramycin and carbamoyltobramycin)*	Biovet®, Huvepharma®
Δ*aprK*	*Derivative of Streptoalloteichus tenebrarius 2444 in which the gene aprK (putative NDP-octodiose synthase) was replaced by an erythromycin resistance cassette*	This study
Δ*aprK*_1-17L	*Derivative of ΔaprK carrying an additional copy of the tobramycin biosynthetic gene cluster (BGC)*	This study
Streptomyces coelicolor M1146	*Super host for heterologous BGC expression, Δact, Δred, Δcpk, Δcda*	[6]
Streptomyces coelicolor M1146_1-17L	*Streptomyces coelicolor M1146 containing the tobramycin BGC*	This study
Streptomyces coelicolor M1152	*Derivative of Streptomyces coelicolor M1146 with rpoB[C1298T]*	[6]
Streptomyces coelicolor M1152_1-17L	*Streptomyces coelicolor M1152 containing the tobramycin BGC*	This study
Streptomyces coelicolor M1154	*Derivative of Streptomyces coelicolor M1152 with rpsL[A262G]*	[6]
Streptomyces coelicolor M1154_1-17L	*Streptomyces coelicolor M1154 containing the tobramycin BGC*	This study
Streptomyces lividans TK24	*Low protease activity, accepts methylated DNA*	[7,8]

**Strain**	**Specifications**	**Reference or source**
Streptomyces lividans TK24_1-17L	*Streptomyces lividans containing the tobramycin BGC*	This study
Streptomyces albus	*Wild type*	[9]

Table S3: Media

Medium	Components	g/1 L H_2O_{dest}
LB Medium (Lennox), ready for use (Roth)	LB-Broth	20
LB Agar (Lennox), ready for use (Roth)	LB-Broth<br>Agar	20<br>16
TSB Medium (Bacto™ Tryptic Soy Broth Soybean-Casein Digest Medium, ready for use; Becton, Dickinson and company)	TSB-Broth	30
TSB Agar (Bacto™ Tryptic Soy Broth Soybean-Casein Digest Medium, ready for use; Becton, Dickinson and company)	TSB-Broth<br>Agar	30<br>16
MS (modified)	Mannitol<br>Soy flour (full fat)<br>Soy flour (low fat)<br>$MgCl_2$<br>(Tap water<br>Distilled water)	20<br>20<br>1<br>2<br>(300 ml<br>700 ml)
MS Agar (modified)	Mannitol<br>Soy flour (full fat)<br>Soy flour (low fat)<br>$MgCl_2$<br>Agar<br>(Tap water<br>Distilled water)	20<br>20<br>1<br>2<br>16<br>(300 ml<br>700 ml)
FC	Soy flour<br>NH_4Cl<br>$MgSO_4$<br>Soybean oil<br>**pH 6.2** (with 20 % NaOH)<br>$CaCO_3$ (added at pH 6.2)<br>D-Glucose (separately sterilized and supplemented)	35<br>6<br>11<br>15<br><br>7<br>40
M9	D-Xylose<br>Soy flour<br>$CaCO_3$<br>NaCl	5<br>10<br>1<br>5

M10	Starch	30
	D-Glucose	5
	Corn Steep Solids	5
	Soy flour	30
	$CaCO_3$	7
	$CoCl_2$	0.13
M11	Dextrin	50
	D-Glucose	5
	Soy flour	35
	$CaCO_3$	7
	$CoCl_2$	0.0013
M12	Lactose	10
	Soy flour	10
	$CaCO_3$	1
	NaCl	5
M14	D-Glucose	10
	Soy flour	10
	NaCl	5
	$CaCO_3$	1
M15	D-Glucose	20
	Beef extract	10
	Yeast extract	10
	$(NH_4)_2SO_4$	3
	$CaCO_3$	0.02
	K_2HPO_4	0.5
	NaCl	2.5
	$FeSO_4$	0.2
	$CoCl_2$	0.02
M16	Dextrin	10
	Corn Steep Solids	20
	Soy flour	10
	$CaCO_3$	2
	K_2HPO_4	2
	NaCl	5
2xYT Medium	Yeast extract	10
	NaCl	5
	Tryptone	16
Electroporation Medium	Saccharose	171
	$MgCl_2$	0.095
CRM Medium	Glucose	100
	Sucrose	103
	$MgCl_2$	10.12
	Tryptone	16
	Yeast extract	5

CRM Agar	Glucose<br>Sucrose<br>$MgCl_2$<br>Tryptone<br>Yeast extract<br>Agar	100<br>103<br>10.12<br>16<br>5<br>16

Table S4: Antibiotics

**Antibiotic**	**Working concentration for *E.coli***	**Working concentration for actinomycetes**
Apramycin	100 μg ml^{-1}	-
Chloramphenicol	100 μg ml^{-1}	-
Trimethoprim	10 μg ml^{-1}	-
Erythromycin	-	100-150 μg ml^{-1}
Thiostrepton	-	150-200 μg ml^{-1}
Nalidixic acid	-	50 μg ml^{-1}

Table S5: Primers and PCRs

The PCR reactions were conducted using the Q5® High-Fidelity PCR Kit.

**Primer name**	**Sequence (5' - 3')**	**Product and product size**	**PCR-Program**
113down_hldE3.FOR	GAGCTCGGTACCCGGGGAT CCTCGACGCGCTGGACGGCC	Right flanking region of *aprK* gene, 1242 bp	1. 98°C, 1 min<br>2. 98°C, 0:15 min<br>3. 72°C, 0:30 min<br>4. 72°C, 0:30 min<br>5. 72°C, 3:00 min<br>6. 4°C, ∞<br>(Steps 2-4 × 32)
114down_hldE3.REV	CGTCTCGATGTCTAGACTGC TGGAGTCGGGGAGGTAACG		
115up_hldE3.REV	CAGTGATAAGCATTACCCTG TTATCCCTACATGCCCCGGC TGCGGTC	Left flanking region of *aprK* gene, 1237 bp	1. 98°C, 1 min<br>2. 98°C, 0:15 min<br>3. 72°C, 0:30 min<br>4. 72°C, 0:30 min<br>5. 72°C, 3:00 min<br>6. 4°C, ∞<br>(Steps 2-4 × 32)
116up_hldE3.FOR	GCAGTCTAGACATCGAGAC GACTTTTGGCGAAGTAGGC		
117_Thiocas.REV	AAAGTCGTCTCGATGTCTAG AAATCTAGAGGCGAATACTT CATATGCGG	Thiostrepton resistance cassette, 1236 bp	1. 98°C, 1 min<br>2. 98°C, 0:15 min<br>3. 68°C, 0:30 min

Primer name	Sequence (5' - 3')	Product and product size	PCR-Program
118_Thiocas.FOR	CCCCGACTCCAGCAGTCTAGATCGATACCGTCGATCCTACCAACCG		4. 72°C, 0:30 min<br>5. 72°C, 3:00 min<br>6. 4°C, ∞<br>(Steps 2-4 × 32)
157_ermEery_SP1_F	AAGTCGTCTCGATGTCTAGATCTTCCGCTGTACCAGCCCG	Erythromycin resistance cassette, 1719 bp	1. 98°C, 1 min<br>2. 98°C, 0:15 min<br>3. 70°C, 0:30 min<br>4. 72°C, 0:30 min<br>5. 72°C, 3:00 min<br>6. 4°C, ∞<br>(Steps 2-4 × 33)
158_ermEery_SP1_R	GCGTGCACCTGCGAAGATCTACGACCGAGCGCAGCGA		
204_1EryPCR_F	GCTCGCCGGTTCGTCCGTCGT	Internal fragment of the erythromycin resistance cassette, 809 bp	1. 98°C, 1 min<br>2. 98°C, 0:15 min<br>3. 70°C, 0:30 min<br>4. 72°C, 0:30 min<br>5. 72°C, 3:00 min<br>6. 4°C, ∞<br>(Steps 2-4 × 33)
205_1EryPCR_R	CGTCGAAGGGCTGCTCACCAGGG		
234_C-hldE3.F	TCGTTGAGCAGCACGTAGTCCAC	Internal fragment of the *aprK* gene, 224 bp	1. 98°C, 0:50 min<br>2. 98°C, 0:10 min<br>3. 69°C, 0:30 min<br>4. 72°C, 0:15 min<br>5. 72°C, 6:00 min<br>6. 4°C, ∞<br>(Steps 2-4 × 28)
235_C-hldE3.R	AGCAGCGGAACCACGCTC		
P1_T_C_Fw	TACCAGGACGGGCTGCCGCTCACG	Internal fragment of the central region of the tobramycin BGC (screening for tobramycin cluster in PAC library), 569 bp	1. 98°C, 0:30 min<br>2. 98°C, 0:10 min<br>3. (deleted)<br>4. 72°C, 0:15 min<br>5. 72°C, 2:00 min<br>6. 4°C, ∞<br>(Steps 2-4 × 29)
P2_T_C_Rev	CTCGTCGGCCAGTCGGAGCAGGCC		
P3_T_L_Fw	GGCGTGGTGTTGAAGCGGGCGTTG	Internal fragment of the left region of the tobramycin BGC (screening for tobramycin cluster in PAC library), 629 bp	1. 98°C, 0:30 min<br>2. 98°C, 0:10 min<br>3. (deleted)<br>4. 72°C, 0:15 min<br>5. 72°C, 2:00 min<br>6. 4°C, ∞<br>(Steps 2-4 × 29)
P4_T_L_Rev	ATCGGCCTAAAGGTCGACTCCCGG		
P5_T_R_Fw	GACTCGTCAGGCGAGACTCATCGG	Internal fragment of the right region of the tobramycin BGC (screening for tobramycin cluster in PAC library), 640 bp	1. 98°C, 0:30 min<br>2. 98°C, 0:10 min<br>3. (deleted)<br>4. 72°C, 0:15 min<br>5. 72°C, 2:00 min<br>6. 4°C, ∞<br>(Steps 2-4 × 29)
P6_T_R_Rev	TCACGGTCGACGCGTCGTACAGC		
P7_A_C_Fw	TCGAGCGCCTTCCTGAGGAAGGCA	Internal fragment of the central	1. 98°C, 0:30 min<br>2. 98°C, 0:10 min

Primer name	Sequence (5' - 3')	Product and product size	PCR-Program
P8_A_C_Rev	TGAACGCGATGACCAGGAA CCTGG	region of the apramycin BGC (screening for tobramycin cluster in PAC library), 587 bp	3. (deleted)<br>4. 72°C, 0:15 min<br>5. 72°C, 2:00 min<br>6. 4°C, ∞<br>(Steps 2-4 × 29)
P9_A_L_Fw	TCCTCGCTGGAGGCGGTGTT CGCCGA	Internal fragment of the left region of the apramycin BGC (screening for tobramycin cluster in PAC library), 544 bp	1. 98°C, 0:30 min<br>2. 98°C, 0:10 min<br>3. (deleted)<br>4. 72°C, 0:15 min<br>5. 72°C, 2:00 min<br>6. 4°C, ∞<br>(Steps 2-4 × 29)
P10_A_L_Rev	GCGGGCGACCTTGTACGGAT GCTT		
P11_A_R_Fw	ATCTCCCGAGCGTCCATCTC GCG	Internal fragment of the right region of the apramycin BGC (screening for tobramycin cluster in PAC library), 656 bp	1. 98°C, 0:30 min<br>2. 98°C, 0:10 min<br>3. (deleted)<br>4. 72°C, 0:15 min<br>5. 72°C, 2:00 min<br>6. 4°C, ∞<br>(Steps 2-4 × 29)
P12_A_R_Rev	AGACCTTCGATGCGTGACCG AAGC		
277_pESACint_F	AGTAGTGCCCCAACTGGGGT AACC	Internal fragment of the PAC 1-17L (which contains the tobramycin BGC), 707 bp	1. 98°C, 0:30 min<br>2. 98°C, 0:10 min<br>3. (deleted)<br>4. 72°C, 0:30 min<br>5. 72°C, 2:00 min<br>6. 4°C, ∞<br>(Steps 2-4 × 30)
278_pESACint_R	GTCCGGTAAGGGGAGTGGT CGAGT		
279_pESAC_plas_F	CACGCCATGATATGCTGCAG ATCCC	Internal fragment of the PAC 1-17L (which contains the tobramycin BGC), 816 bp	1. 98°C, 0:30 min<br>2. 98°C, 0:10 min<br>3. (deleted)<br>4. 72°C, 0:30 min<br>5. 72°C, 2:00 min<br>6. 4°C, ∞<br>(Steps 2-4 × 30)
280_pESAC_plas_R	TTAACTCTATGATACCGAGG GCGCCGT		
281_pESAC_traJ_F	GCTCTTCTTGATGGAGCGC[illegible] TGGG	Internal fragment of the PAC 1-17L (which contains the tobramycin BGC), 614 bp	1. 98°C, 0:30 min<br>2. 98°C, 0:10 min<br>3. (deleted)<br>4. 72°C, 0:30 min<br>5. 72°C, 2:00 min<br>6. 4°C, ∞<br>(Steps 2-4 × 30)
282_pESAC_traJ_R	TTGGTGTATCCAACGGCGTC AGCC		
283_pESAC_uplox_F	GGATGCGCAAGGCGATGGA ACTCA	Internal fragment of the PAC 1-17L (which contains the tobramycin BGC), 822 bp	1. 98°C, 0:30 min<br>2. 98°C, 0:10 min<br>3. (deleted)<br>4. 72°C, 0:30 min<br>5. 72°C, 2:00 min<br>6. 4°C, ∞<br>(Steps 2-4 × 30)
284_pESAC_uplox_R	CATCGACTCCTCGATCGTCA ACCAG		

TTGGTGTATCCAACGGCGTGAGCC

2. Supplementary results

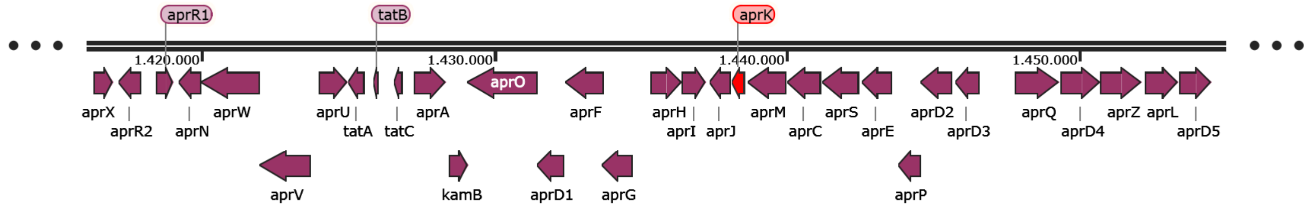

Figure S1: Putative apramycin biosynthetic gene cluster (BGC) from *Streptoalloteichus tenebrarius* 2444. (https://www.dropbox.com/sh/jwxxdzh26worn7g/AADH7xfjNLeUB_V9MMBTYuuWa?dl=0). The putative BGC encompasses a region of approximately 38 kb. The *aprK* gene encoding the NDP-octose synthase is highlighted (in red). The *aprK* gene was inactivated in *S. tenebrarius* 2444 in this study.

Table S6: Analysis of the gene products of the genomic area covering the apramycin biosynthetic gene cluster of *Streptoalloteichus tenebrarius* 2444. The table presents the results of a BlastP [10,11] analysis.

Gene	Protein	BlastP-Hits (Accession)	Precent Identity (BlastP)	Putative Function	References for functional characterization
aprX	AprX	AprX (CAF33026.1)	100 %	putative lipoprotein, *S. tenebrarius*	-
		(SHG39497.1)	66.51%	Uncharacterized conserved protein, DUF305 family, *Streptoalloteichus hindustanus*	-
aprR2	AprR2	AprR2 (CAF33027.1)	100 %	putative transcriptional regulator of AraC family, *S. tenebrarius*	-
aprR1	AprR1	AprR1 (CAF33028.1)	100%	putative AraC-family transcriptional regulator, *S. tenebrarius*	-
aprN	AprN	AprN (CAF33029.1)	100 %	putative aminoglycoside N-acetylhexosaminyl deacetylase or alpha-ketoglutaramate amidase,	-

Gene	Protein	BlastP-Hits (Accession)	Precent Identity (BlastP)	Putative Function	References for functional characterization
				S. tenebrarius	
aprW	AprW	AprW (CAF33030.1)	100%	putative ABC-type aminoglycoside exporter, *S. tenebrarius*	-
aprV	AprV	AprV (CAF33031.1)	100%	putative ABC-type aminoglycoside exporter, *S. tenebrarius*	-
aprU	AprU	AprU (CAF33032.1)	100%	aminoglycoside phosphotransferase, *S. tenebrarius*	[12]
tatA	TatA	TatA (CAF33033.1)	100%	putative transposase, *S. tenebrarius*	-
tatB	TatB	TatB (CAF33034.1)	100%	putative transposase (fragment), *S. tenebrarius*	-
tatC	TatC	TatC (CAF33035.1)	100%	putative transposase (fragment), *S. tenebrarius*	-
aprA	AprA	AprA (CAF33036.1) (AAN05728.1)	100%	apramycin biosynthesis protein, *S. tenebrarius*	-
kamB	KamB	KamB (WP_063964000.1)	100%	16S rRNA (adenine(1408)-N(1))-methyltransferase KamB, *S. tenebrarius*	[13]
aprO	AprO	AprO (CAF33038.1)	100%	putative oligosaccharide phosphorylase or hydrolase, *S. tenebrarius*	
aprD1	AprD1	AprD1 (CAF33039.1)	100%	putative apramycin biosynthetic oxidoreductase 1, *S. tenebrarius*	-
		(WP_073487331.1)	80.13%	NAD-dependent epimerase/dehydratase family protein, *S. hindustanus*	[14]

Gene	Protein	BlastP-Hits (Accession)	Precent Identity (BlastP)	Putative Function	References for functional characterization
aprF	AprF	AprF (CAF33040.1)	99.76%	Unknown (pentapeptide repeats), *S. tenebrarius*	-
		AprF (AAQ99278.1)	99.75%	AprF, *S. tenebrarius*	-
aprG	AprG	AprG (AAQ99277.1)	100%	putative apramycin biosynthesis protein (hydrolase?), *S. tenebrarius*	-
aprH	AprH	AprH (CAF33042.1)	100%	putative glycosyltransferase, *S. tenebrarius*	[15]
aprI	AprI	AprI (CAF33043.1)	100%	putative oxidase (apramycin biosynthesis N-methyltransferase), *S. tenebrarius*	-
aprJ	AprJ	AprJ (CAF33044.1)	92.05%	putative phosphosugar mutase, *S. tenebrarius*	-
		(WP_073487319.1)	81.47%	HAD family phosphatase, *S. hindustanus*	-
aprK	AprK	AprK (CAF33045.1)	100%	NDP-octose synthase, *S. tenebrarius*	This study, [16]
aprM	AprM	AprM (CAF33046.1)	95.66%	putative glycosyltransferase, *S. tenebrarius*	-
aprC	AprC	AprC (CAF33047.1)	100%	2-deoxy-scyllo-inosose synthase, *S. tenebrarius*	-
aprS	AprS	AprS (CAF33048.1)	100%	putative ketocyclitol aminotransferase, *S. tenebrarius*	-
aprE	AprE	AprE (CAF33049.1)	100%	putative 3-amino-2,3-dideoxy-scyllo-inositol 1-dehydrogenase, *S. tenebrarius*	-
aprP	AprP	AprP (CAF33050.1)	100%	creatinine amidohydrolase, *S. tenebrarius*	[12]

Gene	Protein	BlastP-Hits (Accession)	Precent Identity (BlastP)	Putative Function	References for functional characterization
aprD2	AprD2	AprD2 (CAF33051.1)	100%	putative apramycin biosynthetic oxidoreductase 2 (UDP-N-acetylglucosamine 4,6 dehydratase), *S. tenebrarius*	-
aprD3	AprD3	AprD3 (CAF33052.1)	100%	NADPH-dependent reductase, *S. tenebrarius*	[17-19]
aprY(?)	AprY	-	100%	Unknown (*S. tenebrarius* genomic region of the apramycin biosynthesis cluster, type strain DSM 40477T (AJ629123.1)	-
aprQ	AprQ	AprQ (CAF33053.1)	100%	aminoglycoside 6'-dehydrogenase, *S. tenebrarius*	[19-21]
aprD4	AprD4	AprD4 (6FD2_A)	100%	radical S-adenosyl-L-methionine (SAM) enzyme (dehydration of paromamine), *S. tenebrarius*	[17,18,20]
aprZ	AprZ	AprZ (CAF33055.1)	100%	alkaline phosphatase, *S. tenebrarius*	[12]
aprL	AprL	AprL (CAF33056.1)	100%	putative apramycin biosynthetic aminotransferase, *S. tenebrarius*	-
aprD5	AprD5	AprD5 (CAF33057.1)	100%	putative apramycin biosynthetic oxidoreductase 5 (UDP-glucose 4-epimerase), *S. tenebrarius*	-

A

putative phosphosugar mutase [Streptoalloteichus tenebrarius]

Sequence ID: CAF33044.1 **Length:** 238 **Number of Matches:** 1

Range 1: 1 to 238 GenPept Graphics ▼ Next Match ▲ Previous Match

Score	Expect	Method	Identities	Positives	Gaps
394 bits(1013)	4e-137	Compositional matrix adjust.	220/239(92%)	221/239(92%)	1/239(0%)

```
Query  1    MSRAATGEGTLVVLDIDGTLLDTPHLPAWRRGLARVLGDHAPDRSAEISVEQYHRHVAGR  60
            MSRAATGEGTLVVLDIDGTLLDTPHLPAWRRGLARVLGDHAPDRSAEISVEQYHRHVAGR
Sbjct  1    MSRAATGEGTLVVLDIDGTLLDTPHLPAWRRGLARVLGDHAPDRSAEISVEQYHRHVAGR  60

Query  61   PRQVGAAAALGIAGLDPTPELVEELAVVKQELFLEQAEETVLFPDARDFLDAAAREGTPV  120
            PRQVGAAAALGIAGLDPTPELVEELAVVKQELFLEQAEETVLFPDARDFLDAAAREGTPV
Sbjct  61   PRQVGAAAALGIAGLDPTPELVEELAVVKQELFLEQAEETVLFPDARDFLDAAAREGTPV  120

Query  121  AFCTASRNAGELLAKRLPGLDGGDWLLDRLHQSLGPHGHYGDVPRPEALRRVARAWNWSP  180
            AFCTASRNAGELLAKRLPGLDGGDWLLDRLHQSLGPHGHYGDVPRPEALRRVARAWNWSP
Sbjct  121  AFCTASRNAGELLAKRLPGLDGGDWLLDRLHQSLGPHGHYGDVPRPEALRRVARAWNWSP  180

Query  181  DRCVLVDDALSGVLAGQEVGMRPVLLDRFGLGVAAPGCPVVATLDELRPLPGGLRLPVA  239
            DRCVLVDDALSGVLAGQEVGMRPVLLDRFGL     G P      E+      LRLPVA
Sbjct  181  DRCVLVDDALSGVLAGQEVGMRPVLLDRFGLVSPRRGVPWSPPSTEVAD-SRRLRLPVA  238
```

B

putative glycosyltransferase [Streptoalloteichus tenebrarius]

Sequence ID: CAF33046.1 Length: 438 Number of Matches: 1

```
Range 1: 1 to 438 GenPept Graphics                    ▼ Next Match ▲ Previous Match

Score          Expect Method                        Identities     Positives      Gaps
810 bits(2093) 0.0    Compositional matrix adjust.  419/438(96%)   422/438(96%)   1/438(0%)

Query  3    VLRLTPFFHHDCVDSWPAEFDSVGGMQVQILRLSRQLAQRGVRQEVFTVGFPGLPRVRED  62
            +LRLTPFFHHDCVDSWPAEFDSVGGMQVQILRLSRQLAQRGVRQEVFTVGFPGLPRVRED
Sbjct  1    MLRLTPFFHHDCVDSWPAEFDSVGGMQVQILRLSRQLAQRGVRQEVFTVGFPGLPRVRED  60

Query  63   SPGLVVRITRAPMPRLRSELTGLVGLNLAWFLGAMAECLRRRRGPLPDLIQVHGDGQLWA  122
            SPGLVVRITRAPMPRLRSELTGLVGLNLAWFLGAMAECLRRRRGPLPDLIQVHGDGQLWA
Sbjct  61   SPGLVVRITRAPMPRLRSELTGLVGLNLAWFLGAMAECLRRRRGPLPDLIQVHGDGQLWA  120

Query  123  LLAGPLASAILRRPYSLVLHCSRLGVYQPMSRYDRWQHRFVAAVERWAVRRASGVCALTT  182
            LLAGPLASAILRRPYSLVLHCSRLGVYQPMSRYDRWQHRFVAAVERWAVRRASGVCALTT
Sbjct  121  LLAGPLASAILRRPYSLVLHCSRLGVYQPMSRYDRWQHRFVAAVERWAVRRASGVCALTT  180

Query  183  RTADVVRKALRPHQVRVDVVPDSVDPDPPAHSGVPVADRLRAAGLPPDARVVGYVGRVAH  242
            RTADVVRKALRPHQVRVDVVPDSVDPDPPAHSGVPVADRLRAAGLPPDARVVGYVGRVAH
Sbjct  181  RTADVVRKALRPHQVRVDVVPDSVDPDPPAHSGVPVADRLRAAGLPPDARVVGYVGRVAH  240

Query  243  EKGWSHFVDVAERLAGGPAGERVVFLVVGDGPQRPRMAERVAAAGLADRFVFTGFLPNQD  302
            EKGWSHFVDVAERLAGGPAGERVVFLVVGDGPQRPRMAERVAAAGLADRFVFTGFLPNQD
Sbjct  241  EKGWSHFVDVAERLAGGPAGERVVFLVVGDGPQRPRMAERVAAAGLADRFVFTGFLPNQD  300

Query  303  IPLTMGGIDVLVMPSVHEELGGSAIEAMVLGVPVVAYGVGGLRDTVGRVTPSLAVRPQDV  362
            IPLTMGGIDVLVMPSVHEELGGSAIEAMVLGVPVV YGVGGL DTVGRVTPSLAVRPQDV
Sbjct  301  IPLTMGGIDVLVMPSVHEELGGSAIEAMVLGVPVVDYGVGGLTDTVGRVTPSLAVRPQDV  360

Query  363  GALTDAVRDVLARTDEYRA-QVRAGRPWLEENYGDGVGVTRTVAHYHRILAGGRGGVTAG  421
            GALT       +      A +VRAGRPWLEENYGDGVGVTRTVAHYHRILAGGRGGVTAG
Sbjct  361  GALTLTPSATSSPGPTSTAPRVRAGRPWLEENYGDGVGVTRTVAHYHRILAGGRGGVTAG  420

Query  422  AGLPDDPARAGRSPASGR  439
            AGLPDDPARAGRSPASGR
Sbjct  421  AGLPDDPARAGRSPASGR  438
```

Figure S2: BlastP analysis of AprJ and AprM. (**A**) The alignment shows mutation in the putative phosphosugar mutase AprJ of *Streptoalloteichus tenebrarius* 2444 PS compared to the amino acid sequence of the hit (CAF33044.1) from the The National Center for Biotechnology Information (NCBI) database. (**B**) The alignment shows mutation in the putative glycosyltransferase AprM of *S. tenebrarius* 2444 PS compared to the amino acid sequence of the hit (CAF33046.1) from the NCBI database.

A

putative aminoglycoside 4-glucosaminyltransferase, TobM1 [Streptoalloteichus tenebrarius]

Sequence ID: CAH18562.1 Length: 416 Number of Matches: 1

Range 1: 1 to 416 GenPept Graphics ▼ Next Match ▲ Previous Match

Score	Expect	Method	Identities	Positives	Gaps
813 bits(2101)	0.0	Compositional matrix adjust.	415/416(99%)	416/416(100%)	0/416(0%)

```
Query  6    VRVLRLTPFFHHDCVTSWPAEFDAVGGMQLQILRLSRELARRGVRQQVLTLGFPGLPRVR  65
            +RVLRLTPFFHHDCVTSWPAEFDAVGGMQLQILRLSRELARRGVRQQVLTLGFPGLPRVR
Sbjct  1    MRVLRLTPFFHHDCVTSWPAEFDAVGGMQLQILRLSRELARRGVRQQVLTLGFPGLPRVR  60

Query  66   VDSPNLVVRITRAPLPRLRSELTGLVGLNQAWLVAALAACVRLRRTWRPDLVHVHADGQL  125
            VDSPNLVVRITRAPLPRLRSELTGLVGLNQAWLVAALAACVRLRRTWRPDLVHVHADGQL
Sbjct  61   VDSPNLVVRITRAPLPRLRSELTGLVGLNQAWLVAALAACVRLRRTWRPDLVHVHADGQL  120

Query  126  WALLAGPAASRVLGVPYCVTLHCSRLSVYQPMSWIDQLQHRLVVAAEKWALRGASGVSTL  185
            WALLAGPAASRVLGVPYCVTLHCSRLSVYQPMSWIDQLQHRLVVAAEKWALRGASGVSTL
Sbjct  121  WALLAGPAASRVLGVPYCVTLHCSRLSVYQPMSWIDQLQHRLVVAAEKWALRGASGVSTL  180

Query  186  TDRTASVVASALGVGAEDVDVVPDSVDTSSTVDRAEGRVLLEKLGVPSDHEAVGYVGRVA  245
            TDRTASVVASALGVGAEDVDVVPDSVDTSSTVDRAEGRVLLEKLGVPSDHEAVGYVGRVA
Sbjct  181  TDRTASVVASALGVGAEDVDVVPDSVDTSSTVDRAEGRVLLEKLGVPSDHEAVGYVGRVA  240

Query  246  HEKGWPDLVRVAGALSDRKATFLVVGDGPQSGRMRDEVAAAGLSDRFLFTGFLPHHDIPA  305
            HEKGWPDLVRVAGALSDRKATFLVVGDGPQSGRMRDEVAAAGLSDRFLFTGFLPHHDIPA
Sbjct  241  HEKGWPDLVRVAGALSDRKATFLVVGDGPQSGRMRDEVAAAGLSDRFLFTGFLPHHDIPA  300

Query  306  VMAGLDVLVMPSRHEELGGSALEAMLAGTPVAAYAVGGLRDTVGHVTPSLLVPPGDVTAL  365
            VMAGLDVLVMPSRHEELGGSALEAMLAGTPVAAYAVGGLRDTVGHVTPSLLVPPGDVTAL
Sbjct  301  VMAGLDVLVMPSRHEELGGSALEAMLAGTPVAAYAVGGLRDTVGHVTPSLLVPPGDVTAL  360

Query  366  AEAVRGVLDDPRPHRDQVAAGRSWLTDLFDGGAAARRVIAHYERVLSGTARLRPQE  421
            AEAVRGVLDDPRPHRDQVAAGRSWLTDLFDGGAAARRVIAHYERVLSGTARLRPQE
Sbjct  361  AEAVRGVLDDPRPHRDQVAAGRSWLTDLFDGGAAARRVIAHYERVLSGTARLRPQE  416
```

B

MGRGPVRVLRLTPFFHHDCVTSWPAEFDAVGGMQLQILRLSRELARRGVRQQVLTLGFPG
LPRVRVDSPNLVVRITRAPLPRLRSELTGLVGLNQAWLVAALAACVRLRRTWRPDLVHVHA
DGQLWALLAGPAASRVLGVPYCVTLHCSRLSVYQPMSWIDQLQHRLVVAAEKWALRGASG
VSTLTDRTASVVASALGVGAEDVDVVPDSVDTSSTVDRAEGRVLLEKLGVPSDHEAVGYVG
RVAHEKGWPDLVRVAGALSDRKATFLVVGDGPQSGRMRDEVAAAGLSDRFLFTGFLPHHD
IPAVMAGLDVLVMPSRHEELGGSALEAMLAGTPVAAYAVGGLRDTVGHVTPSLLVPPGDVT
ALAEAVRGVLDDPRPHRDQVAAGRSWLTDLFDGGAAARRVIAHYERVLSGTARLRPQE*

Figure S3: BlastP analysis of TobM1. (**A**) BlastP alignment of the putative glycosyltransferase TobM1 from *Streptoalloteichus. tenebrarius* 2444 PS and its hit (CAH18562.1) from the The National Center for Biotechnology Information (NCBI) database. (**B**) The amino acid sequence of TobM1, including the extension of the N-terminus (in red).

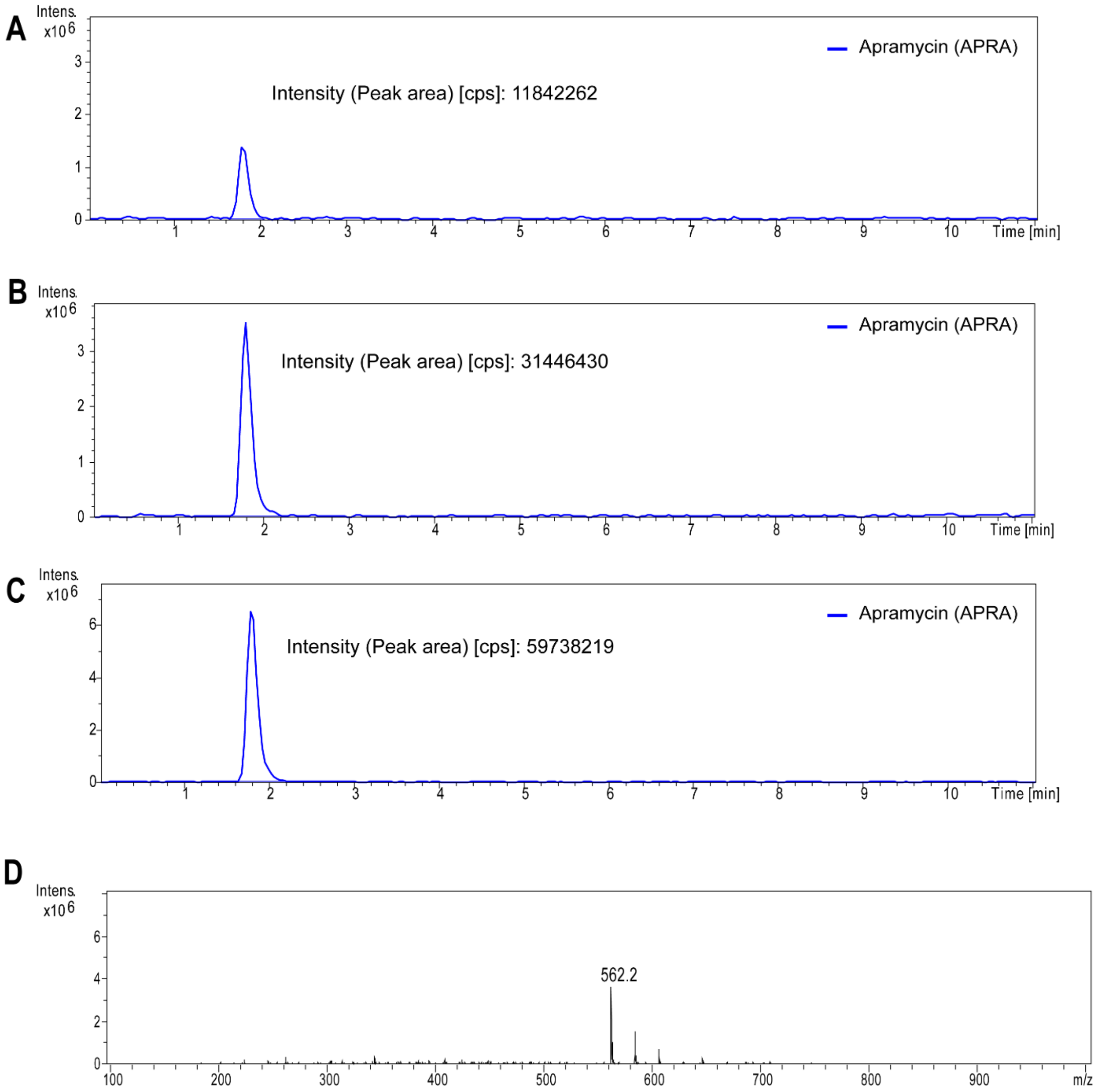

Figure S4. Spectra of the HPLC-MS analysis of the reference compound apramycin (APRA). (**A-C**) Extracted ion chromatogram spectra for increasing concentration of APRA (m/z [M+Na]+=562). (**D**) Mass for the extracted ion chromatogram (m/z [M+Na]+=562).

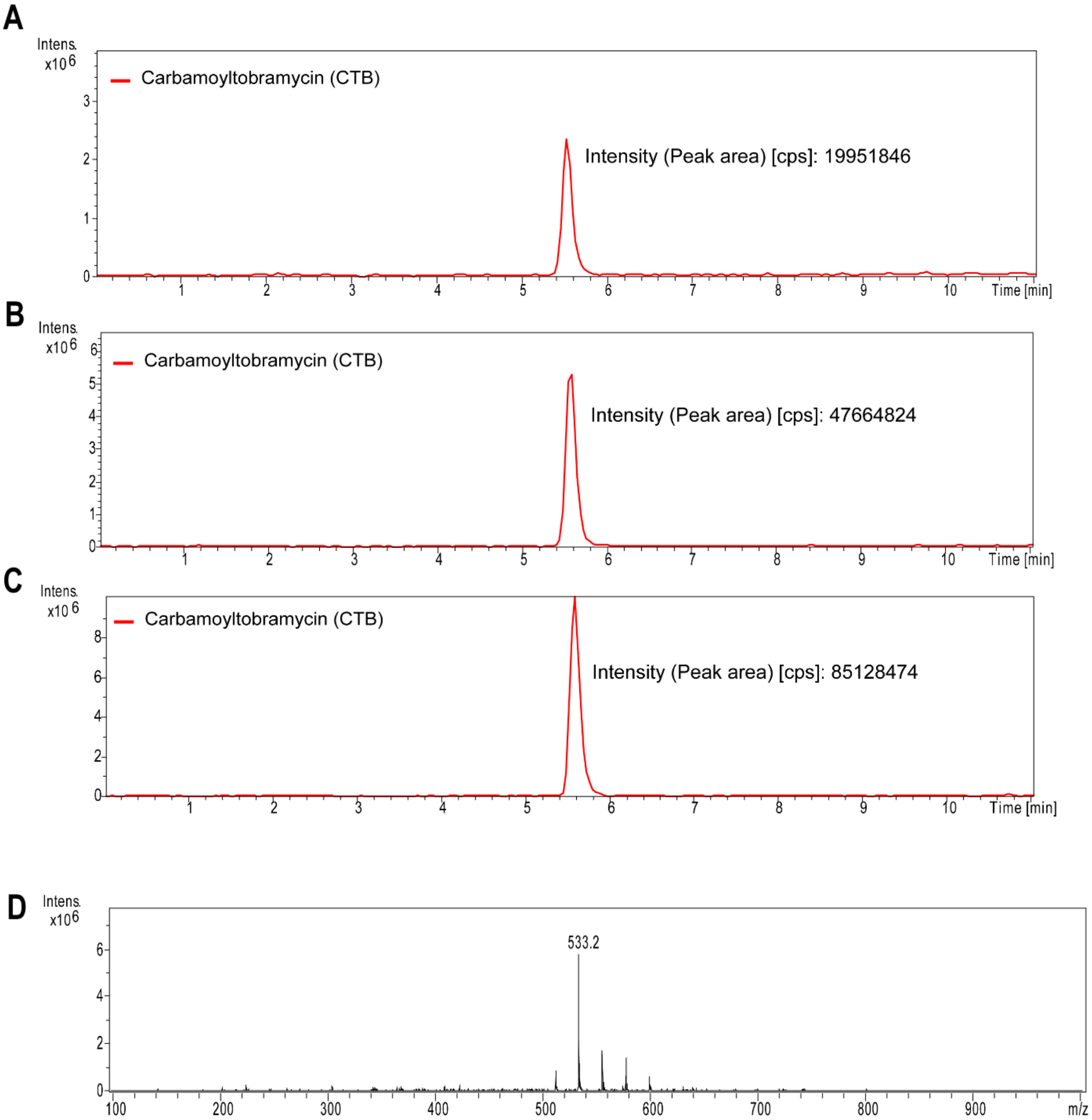

Figure S5. Spectra of the HPLC-MS analysis of the reference compound carbamoyltobramycin (CTB). (**A-C**) Extracted ion chromatogram spectrum for increasing concentration of CTB (m/z [M+Na]+=533). (**D**) Mass for the extracted ion chromatogram (m/z [M+Na]+=533).

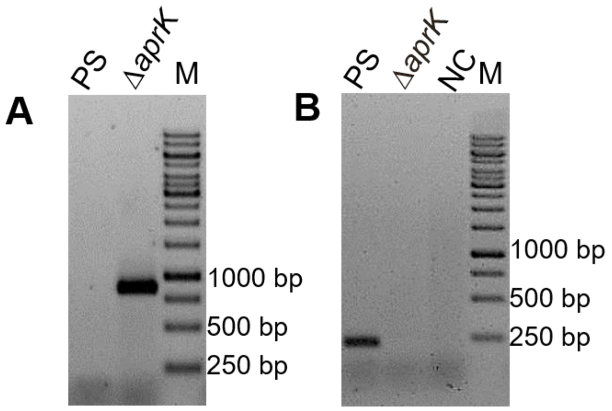

Figure S6. Analysis of screening PCRs for verification of the mutant Δ*aprK*. (**A**) Screening PCR using primers P204 and P205 (no product for the parental strain and PCR product of the size of 809 bp for Δ*aprK*). (**B**) Screening PCR using primers P234 and P235 (PCR product of the size of 224 bp for parental strain and no product for Δ*aprK*). PS: *Streptoalloteichus tenebrarius* 2444 parental strain; Δ*aprK*: gene knockout mutant with inactivated *aprK* gene; NC: negative control (water instead of template); M: Thermo Scientific™ GeneRuler 1 kb DNA Ladder.

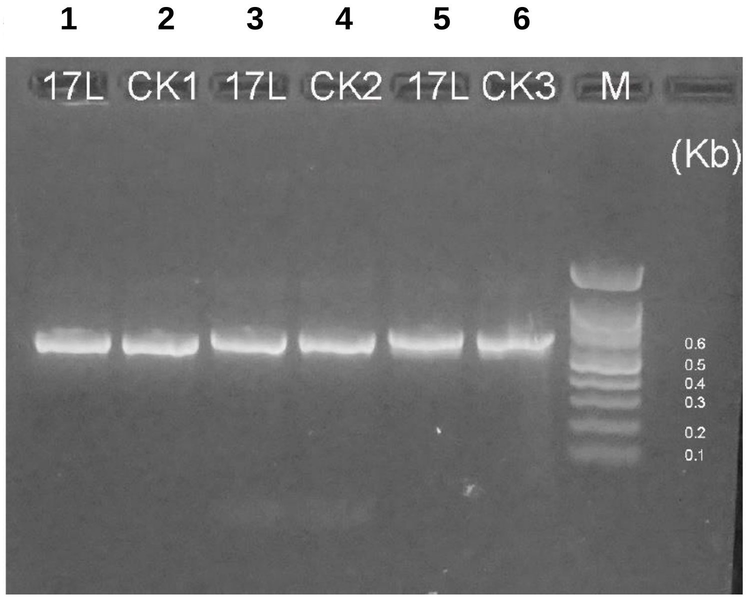

Figure S7: Identification of the PAC containing the tobramycin BGC. The screening PCRs of the PAC library of *Streptoalloteichus tenebrarius* 2444 were analysed in agarose gel electrophoresis. Lane 1: clone 1-17L screened with primer pair P1_T_C_Fw/P2_T_C_Rev. Lane 2: control (gDNA of *S. tenebrarius 2444*) screened with primer pair P1_T_C_Fw/P2_T_C_Rev. Lane 3: clone 1-17L screened with primer pair P3_T_L_Fw/P4_T_L_Rev. Lane 4: control (gDNA of *S. tenebrarius 2444*) screened with primer pair P3_T_L_Fw/P4_T_L_Rev. Lane 5: clone 1-17L screened with primer pair P5_T_R_Fw/P6_T_R_Rev. Lane 6: control (gDNA of *S. tenebrarius 2444*) screened with primer pair P5_T_R_Fw/P6_T_R_Rev.

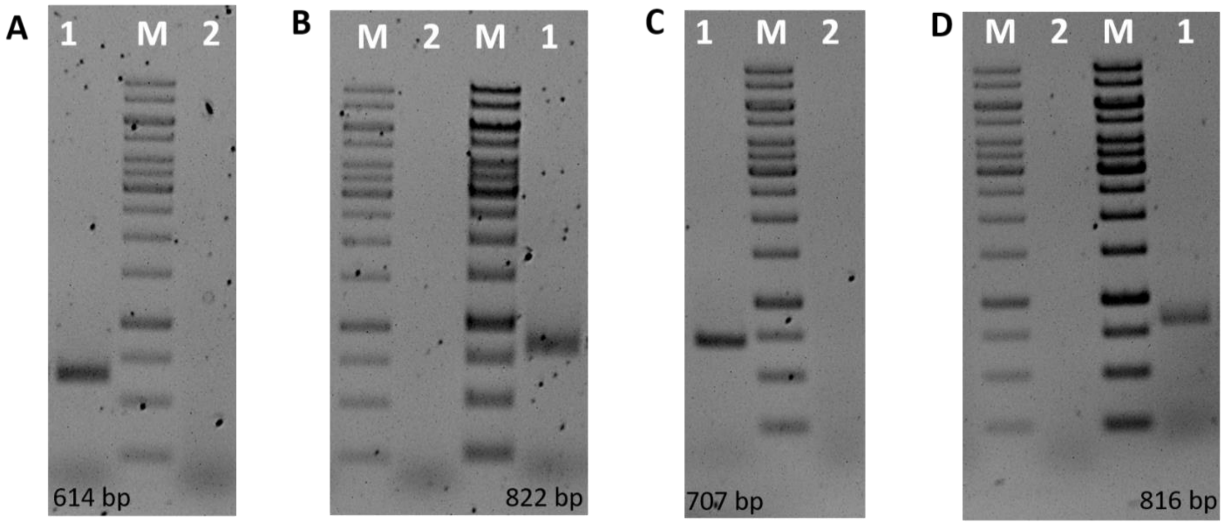

Figure S8: Introduction of the PAC containing the tobramycin BGC (1-17L) into the Δ*aprK* mutant. The screening PCRs of the *ΔaprK*_1-17L mutant and the precursor strain *ΔaprK* were analysed in agarose gel electrophoresis. (**A**) Primer pair 281_pESAC_traJ_F and 282_pESAC_traJ_R (expected: *ΔaprK*: no product; *ΔaprK*_1-17L: 614 bp). (**B**) Primer pair 283_pESAC_uplox_F and 284_pESAC_uplox_R (expected: *ΔaprK*: no product; *ΔaprK*_1-17L: 822 bp). (**C**) Primer pair 277_pESACint_F and 278_pESACint_R (expected: *ΔaprK*: no product; *ΔaprK*_1-17L: 707 bp). (**D**) Primer pair 279_pESAC_plas_F and 280_pESAC_plas_R (expected: *ΔaprK*: no product; *ΔaprK*_1-17L: 816 bp). (1): *ΔaprK*_1-17L mutant. (2) *ΔaprK* (precursor strain). M: Thermo Scientific™ GeneRuler 1 kb DNA Ladder.

HPLC-MS analysis (raw data)

Table S7: Raw data for carbamoyltobramycin (CTB) and apramycin (APRA) production in *Streptoalloteichus tenebrarius* 2444 parental strain (PS) (triplicate, PS_1-3) (Figure 3D).

Sample	Intensity (Peak area) [cps] for apramycin (APRA)	Intensity (Peak area) [cps] for carbamoyltobramycin (CTB)
PS_1	31143937	14668237
PS_2	28778704	16811468
PS_3	29446647	22766086

Table S8: Raw data for the medium screening approach. Carbamoyltobramycin (CTB) and apramycin (APRA) production was determined for *Streptoalloteichus tenebrarius* 2444 parental strain (PS) (triplicates) (Figure 4). *p*-Values are given for comparison between FC medium and each of the screening medium. Null hypothesis (H_0): there is no difference in APRA/CTB

production in the screening medium compared to FC medium. H_1: The APRA production is lower/the CTB production is higher in the screening medium compared to FC medium.

Sample	Intensity* (Peak area) [cps] for (APRA)	Intensity* (Peak area) [cps] for (CTB)	Standard deviation (APRA)	Standard deviation (CTB)	*p*-value (APRA)	*p*-value (CTB)
M9	3438762.67	3362052.67	601541.704	480568.493	< 0.0001	0.0132
M10	1383287	11232205.3	905470.623	846132.356	< 0.0001	0.0546
M11	795544	11697101.7	165411.232	205322.93	0.0003	0.0595
M12	10017348.7	14607243	1883567.7	1116642.8	0.0003	0.1500
M14	2327812	4351340.33	1996130.36	3683010.48	0.0001	0.0065
M15	5039909.67	1634568.33	665108.571	76190.455	< 0.0001	0.0105
M16	694762.667	3352989.33	307099.638	435205.745	0.0003	0.0131
FC	29789762.7	18081930.3	1219376.09	4195753.27	-	-

*Mean value of triplicates (3 independent biological replicates).

Table S9: Raw data for the strain carrying an additional copy of the tobramycin BGC. Apramycin (APRA) and carbamoyltobramycin (CTB) production in the *S. tenebrarius* 2444 parental strain (PS), the Δ*aprK* mutant in *S. tenebrarius* 2444 and the mutant containing an additional copy of the tobramycin BGC (*ΔaprK*_1-17L; the experiment was repeated for three times (A, B, and C)) (Figure 6). *p*-Values are given for comparison between *S. tenebrarius* 2444 PS and the Δ*aprK* mutants (top) and for comparison between Δ*aprK* mutant and the *ΔaprK*_1-17L_A-C mutants (bottom). Null hypothesis (H_0): there is no difference in APRA/CTB production in Δ*aprK* and *ΔaprK*_1-17L compared to *S. tenebrarius* 2444 PS or there is no difference in APRA/CTB production in Δ*aprK* compared to *ΔaprK*_1-17L. H_1: The APRA production is lower/the CTB production is higher when comparing Δ*aprK* and *ΔaprK*_1-17L compared to the PS or when comparing *ΔaprK*_1-17L to Δ*aprK*.

Sample	Intensity* (Peak area) [cps] for (APRA)	Intensity* (Peak area) [cps] for (CTB)	Standard deviation (APRA)	Standard deviation (CTB)	*p*-value (APRA)	*p*-value (CTB)
S. tenebrarius 2444_PS	795544	11697101.67	165411.232	205322.93	-	-
Δ*aprK*	0	7196044	0	2224080.33	0.0071<br>-	0.0366<br>-
Δ*aprK*_1-17L_A	0	40253959	0	14087914.1	0.0071<br>-	0.0362<br>0.0284

Sample	Intensity* (Peak area) [cps] for (APRA)	Intensity* (Peak area) [cps] for (CTB)	Standard deviation (APRA)	Standard deviation (CTB)	*p*-value (APRA)	*p*-value (CTB)
Δ*aprK*_1-17L_B	0	34832502	0	13677497.5	0.0071<br>-	0.0497<br>0.0373
Δ*aprK*_1-17L_C	0	31781629.33	0	14943106	0.0071<br>-	0.0727<br>0.0531

*Mean value of triplicates (3 independent biological replicates).

Table S10: Raw data for cultivation in presence (+) and absence (-) of selection antibiotics. Apramycin (APRA) and carbamoyltobramycin (CTB) production in the Δ*aprK* mutant in *S. tenebrarius* 2444 and the mutant containing an additional copy of the tobramycin BGC (*ΔaprK*_1-17L; the experiment was repeated for three times (A, B, and C)) (Figure 7).

Sample	Intensity (Peak area) [cps] for (CTB),<br>(+) Selection antibiotics	Intensity (Peak area) [cps] for (CTB),<br>(-) Selection antibiotics
Δ*aprK*	27719926	44405392
*ΔaprK*_1-17L_A	43243613	85216257
*ΔaprK*_1-17L_B	62189979	180928786
*ΔaprK*_1-17L_C	34910410	109091274

3. References

1. Myronovskyi, M.; Welle, E.; Fedorenko, V.; Luzhetskyy, A. β-Glucuronidase as a Sensitive and Versatile Reporter in *Actinomycetes*. *Appl Environ Microbiol* **2011**, *77*, 5370-5383, doi:10.1128/AEM.00434-11
2. Myronovskyi, M.; Rosenkränzer, B.; Luzhetskyy, A. Iterative Marker Excision System. *Appl Microbiol Biotechnol* **2014**, *98*, 4557-4570, doi:10.1007/s00253-014-5523-z
3. Muth, G.; Nußbaumer, B.; Wohlleben, W.; Pühler, A. A Vector System with Temperature-Sensitive Replication for Gene Disruption and Mutational Cloning in Streptomycetes. *Mol Gen Genet* **1989**, *219*, 341-348, doi:10.1007/BF00259605.
4. Pelzer, S.; Reichert, W.; Huppert, M.; Heckmann, D.; Wohlleben, W. Cloning and Analysis of a Peptide Synthetase Gene of the Balhimycin Producer *Amycolatopsis mediterranei* DSM5908 and Development of a Gene Disruption/Replacement System. *J Biotech* **1997**, *56*, 115-128, doi:10.1016/s0168-1656(97)00082-5
5. Jones, A.C.; Gust, B.; Kulik, A.; Heide, L.; Buttner, M.J.; Bibb, M.J. Phage p1-Derived Artificial Chromosomes Facilitate Heterologous Expression of the FK506 Gene Cluster. *PLoS One* **2013**, *8*, e69319, doi:10.1371/journal.pone.0069319.
6. Gomez-Escribano, J.P.; Bibb, M.J. Engineering *Streptomyces coelicolor* for Heterologous Expression of Secondary Metabolite Gene Clusters. *Microb Biotechnol* **2011**, *4*, 207-215, doi:10.1111/j.1751-7915.2010.00219.x

7. Hopwood, D.A.; Kieser, T.; Wright, H.M.; Bibb, M.J. Plasmids, Recombination and Chromosome Mapping in *Streptomyces lividans* 66. *Microbiology* **1983**, *129*, 2257-2269, doi:10.1099/00221287-129-7-2257
8. Rückert, C.; Albersmeier, A.; Busche, T.; Jaenicke, S.; Winkler, A.; Friðjónsson, Ó.H.; Hreggviðsson, G.Ó.; Lambert, C.; Badcock, D.; Bernaerts, K. Complete Genome Sequence of *Streptomyces lividans* TK24. *J Biotech* **2015**, *199*, 21-22, doi:10.1016/j.jbiotec.2015.02.004
9. McCarty, M. The Lysis of Group A Hemolytic Streptococci by Extracellular Enzymes of *Streptomyces albus*: I. Production and Fractionation of the Lytic Enzymes. *J Exp Med.* **1952**, *96*, 555-568, doi:10.1084/jem.96.6.555
10. Altschul, S.F.; Gish, W.; Miller, W.; Myers, E.W.; Lipman, D.J. Basic Local Alignment Search Tool. *Mol Biol* **1990**, *215*, 403-410, doi:10.1016/s0022-2836(05)80360-2.
11. States, D.J.; Gish, W. Combined Use of Sequence Similarity and Codon Bias for Coding Region Identification. *J Comput Biol* **1994**, *1*, 39-50, doi:10.1089/cmb.1994.1.39.
12. Zhang, Q.; Chi, H.T.; Wu, L.; Deng, Z.; Yu, Y. Two Cryptic Self-Resistance Mechanisms in *Streptomyces tenebrarius* Reveal Insights into the Biosynthesis of Apramycin. *Angew Chem* **2021**, *133*, 9072-9078, doi:10.1002/anie.202100687
13. Holmes, D.J.; Drocourt, D.; Tiraby, G.; Cundliffe, E. Cloning of an Aminoglycoside-Resistance-Encoding gene, *kamC*, from *Saccharopolyspora hirsuta*: Comparison with *kamB* from *Streptomyces tenebrarius*. *Gene* **1991**, *102*, 19-26, doi:10.1016/0378-1119(91)90532-g
14. Thoden, J.B.; Hegeman, A.D.; Wesenberg, G.; Chapeau, M.C.; Frey, P.A.; Holden, H.M. Structural Analysis of UDP-Sugar Binding to UDP-Galactose 4-Epimerase from *Escherichia coli*. *Biochemistry* **1997**, *36*, 6294-6304, doi:10.1021/bi970025j
15. Hong, W.; Yan, S. Engineering *Streptomyces tenebrarius* to Synthesize Single Component of Carbamoyl Tobramycin. *Lett Appl Microbiol* **2012**, *55*, 33-39, doi:10.1111/j.1472-765X.2012.03254.x
16. Xiao, J.; Li, H.; Wen, S.; Hong, W. Concentrated Biosynthesis of Tobramycin by Genetically Engineered *Streptomyces tenebrarius*. *J Gen App Microbiol* **2014**, *60*, 256-261, doi:10.2323/jgam.60.256
17. Kim, H.J.; LeVieux, J.; Yeh, Y.C.; Liu, H.w. C3′-Deoxygenation of Paromamine Catalyzed by a Radical S-Adenosylmethionine Enzyme: Characterization of the Enzyme AprD4 and Its Reductase Partner AprD3. *Angew Chem Int Edit* **2016**, *55*, 3724-3728, doi:10.1002/anie.201510635
18. Kudo, F.; Tokumitsu, T.; Eguchi, T. Substrate Specificity of Radical S-Adenosyl-l-Methionine Dehydratase AprD4 and Its Partner Reductase AprD3 in the C3′-Deoxygenation of Aminoglycoside Antibiotics. *J Antibiot* **2017**, *70*, 423-428, doi:10.1038/ja.2016.110
19. Lv, M.; Ji, X.; Zhao, J.; Li, Y.; Zhang, C.; Su, L.; Ding, W.; Deng, Z.; Yu, Y.; Zhang, Q. Characterization of a C3 Deoxygenation Pathway Reveals a Key Branch Point in Aminoglycoside Biosynthesis. *J Am Chem Soc* **2016**, *138*, 6427-6435, doi:10.1021/jacs.6b02221
20. Ni, X.; Li, D.; Yang, L.; Huang, T.; Li, H.; Xia, H. Construction of Kanamycin B Overproducing Strain by Genetic Engineering of *Streptomyces tenebrarius*. *Appl Microbiol Biotechnol* **2011**, *89*, 723-731, doi:10.1007/s00253-010-2908-5

21. Wang, J.; Ma, S.; Ding, W.; Chen, T.; Zhang, Q. Mechanistic Study of the Oxidoreductase AprQ Involved in the Biosynthesis of the Aminoglycoside Antibiotic Apramycin. *Chin J Chem* **2021**, doi:10.1002/cjoc.202100070.
